# Supplementary material for: Risk factors for death in Welsh infants with a congenital anomaly
Source: BMJ Paediatr Open. 2021 Dec 28;5(1):e001205. doi: 10.1136/bmjpo-2021-001205 (PMC8718467; doi:10.1136/bmjpo-2021-001205)
Supplement: Supplementary data [file bmjpo-2021-001205supp001.pdf]

Supplementary Table 1: Inclusion of anomaly subgroups.

Subgroup	Classification of anomaly included
All anomalies; all isolated anomalies; and all multiple anomalies	• Nervous system;<br>• Eye, ear, face and neck;<br>• Cardiovascular system;<br>• Respiratory and clefts;<br>• Abdominal wall defects and diaphragmatic hernia;<br>• Upper and lower gastrointestinal;<br>• Genitourinary;<br>• Limb and skeletal;<br>• Blood disorders;<br>• Endocrine and Metabolic;<br>• Neoplasm;<br>• Syndromes and congenital malformation syndromes;<br>• Maternal Infection leading to malformations.
Cardiovascular anomalies	• Congenital heart defects and diseases of the circulatory system.

Ho PSY, *et al. BMJ Paediatrics Open* 2021; 5:e001205. doi: 10.1136/bmjpo-2021-001205

Supplementary Table 2: Description of included variables in the analyses.

Variable	Categorisation
Townsend (as area-based deprivation)	1 (least deprived)[1], 2, 3, 4, 5 (most deprived), and not known/missing.
Maternal ethnicity	White[2], Other (Chinese, Indian, Bangladeshi, Pakistani, Other Asian, Black Caribbean, Black African, Other Black, Mixed, Other), and not known/missing.
Maternal age at birth	≤24, 25-29[2], 30-34, ≥35 years, and not known/missing.
Parity	Nulliparous[2], ≥1, and not known/missing.
Multiple pregnancies	Yes, No[2], and not known/missing.
Maternal smoking	Smoker, Non/ Ex-smoker[2], and not known/missing.
Anomaly in previous pregnancies	Yes, No[2], and not known/missing.
Infant sex	Male, Female[2] and not known/missing.
Infant's birthweight	<2500 (low birthweight), ≥2500[2] g and not known/missing.
Infant's gestational age at birth[2]	<37^{+0} (preterm), ≥37^{+0} [(1)], and not known/missing.
Disease severity (for CHD only)[3]	Less severity[1], moderate severity, most severity, and not known/missing.
Surgery	Performed (or expected) in the first year after birth, not performed or required in the first year after birth[1], and not known/missing.

[1]Reference group. [2]Significant departure of linearity was shown in the effect of gestational age in weeks on infant mortality.[3]Congenital heart defects (CHD) severity category was based on criteria used by Khoshnood et al. (2012): CHD less severe = Ventricular septal defect (VSD), Atrial septal defect (ASD), Pulmonary valve stenosis. CHD moderate severe = Common arterial truncus, Transposition of great vessels, Atrio-ventricular septal defect (AVSD), Tetralogy of Fallot, Pulmonary valve atresia, Aortic valve atresia/ stenosis, Coarctation of aorta, total anomalous pulmonary venous return. CHD most severe = Single ventricle, Tricuspid atresia and stenosis, Ebstein's anomaly, Hypoplastic left heart and Hypoplastic right heart.

Supplementary Table 3: Year of birth added as a covariate in the final model as a sensitivity analysis.

Factors	All anomalies	Isolated anomalies	Multiple anomalies	Cardiovascular anomalies
Ethnicity<br>Other vs. White	2.31 (1.82-2.94)	1.90 (1.18-3.07)	2.24 (1.76-2.84)	1.02 (0.27-3.89)
Parity<br>≥ 1 vs. Nulliparous	1.22 (1.07-1.40)	1.47 (1.15-1.88)	1.22 (1.08-1.40)	
Maternal smoking<br>Smoker vs. Non/Ex smoker	1.19 (1.02-1.39)	1.43 (1.07-1.91)		
Infant sex<br>Female vs. Male	1.28 (1.13-1.45)	1.05 (0.84-1.32)	1.28 (1.12-1.45)	1.23 (0.76-2.02)
Gestational age at birth<br>Preterm vs. Term	4.35 (3.83-4.94)	4.51 (3.59-5.64)	4.39 (3.87-4.99)	3.67 (2.18-6.17)
CHD severity<br>Moderate vs. Less<br>Most vs. Less	n/a	n/a	n/a	18.4 (8.10-41.7)<br>242 (94-620)
Surgery<br>Yes vs. No	0.78 (0.66-0.93)		0.68 (0.58-0.79)	0.31 (0.14-0.70)
Year<br>Per yearly increase (1998-2016)	0.96 (0.95-0.98)	0.98 (0.95-1.00)	0.96 (0.95-0.97)	0.93 (0.88-0.99)

Note: Statistical significance at $p < 0.05$; Adjusted odd ratios with 95% confidence intervals are shown. Empty cell represents variable that was not included in the multivariable analysis. CHD = Congenital heart defects. n/a = not applicable.

Ho PSY, *et al. BMJ Paediatrics Open* 2021; 5:e001205. doi: 10.1136/bmjpo-2021-001205
