# Supplementary material for: Risk factors for death in Welsh infants with a congenital anomaly
Source: BMJ Paediatr Open. 2021 Dec 28;5(1):e001205. doi: 10.1136/bmjpo-2021-001205 (PMC8718467; doi:10.1136/bmjpo-2021-001205)
Supplement: Author's manuscript [file bmjpo-2021-001205.draft_revisions.pdf]

BMJ Paediatrics Open

BMJ Paediatrics Open is committed to open peer review. As part of this commitment we make the peer review history of every article we publish publicly available.

When an article is published we post the peer reviewers' comments and the authors' responses online. We also post the versions of the paper that were used during peer review. These are the versions that the peer review comments apply to.

The versions of the paper that follow are the versions that were submitted during the peer review process. They are not the versions of record or the final published versions. They should not be cited or distributed as the published version of this manuscript.

BMJ Paediatrics Open is an open access journal and the full, final, typeset and author-corrected version of record of the manuscript is available on our site with no access controls, subscription charges or pay-per-view fees (http://bmjpaedsopen.bmj.com).

If you have any questions on BMJ Paediatrics Open's open peer review process please email info.bmjpo@bmj.com

BMJ Paediatrics Open

RISK FACTORS FOR DEATH IN WELSH INFANTS WITH A CONGENITAL ANOMALY.

Journal:	*BMJ Paediatrics Open*
Manuscript ID	bmjpo-2021-001205
Article Type:	Original research
Date Submitted by the Author:	28-Jun-2021
Complete List of Authors:	Ho, Peter ; University of Oxford, National Perinatal Epidemiology Unit, Nuffield Department of Population Health<br>Quigley, Maria; University of Oxford, National Perinatal Epidemiology Unit, Nuffield Department of Population Health; University of Oxford, National Institute for Health Research (NIHR) Policy Research Unit-Maternal Health and Care, National Perinatal Epidemiology Unit, Nuffield Department of Population Health.<br>Tucker, David; Public Health Wales, Congenital Anomaly Register and Information Service for Wales<br>Kurinczuk, Jenny; University of Oxford, National Perinatal Epidemiology Unit, Nuffield Department of Population Health; University of Oxford, National Institute for Health Research (NIHR) Policy Research Unit-Maternal Health and Care, National Perinatal Epidemiology Unit, Nuffield Department of Population Health.
Keywords:	Epidemiology, Mortality

SCHOLARONE™
Manuscripts

I, the Submitting Author has the right to grant and does grant on behalf of all authors of the Work (as defined in the below author licence), an exclusive licence and/or a non-exclusive licence for contributions from authors who are: i) UK Crown employees; ii) where BMJ has agreed a CC-BY licence shall apply, and/or iii) in accordance with the terms applicable for US Federal Government officers or employees acting as part of their official duties; on a worldwide, perpetual, irrevocable, royalty-free basis to BMJ Publishing Group Ltd ("BMJ") its licensees and where the relevant Journal is co-owned by BMJ to the co-owners of the Journal, to publish the Work in this journal and any other BMJ products and to exploit all rights, as set out in our licence.

The Submitting Author accepts and understands that any supply made under these terms is made by BMJ to the Submitting Author unless you are acting as an employee on behalf of your employer or a postgraduate student of an affiliated institution which is paying any applicable article publishing charge ("APC") for Open Access articles. Where the Submitting Author wishes to make the Work available on an Open Access basis (and intends to pay the relevant APC), the terms of reuse of such Open Access shall be governed by a Creative Commons licence – details of these licences and which Creative Commons licence will apply to this Work are set out in our licence referred to above.

Other than as permitted in any relevant BMJ Author's Self Archiving Policies, I confirm this Work has not been accepted for publication elsewhere, is not being considered for publication elsewhere and does not duplicate material already published. I confirm all authors consent to publication of this Work and authorise the granting of this licence.

Confidential: For Review Only

RISK FACTORS FOR DEATH IN WELSH INFANTS WITH A CONGENITAL ANOMALY.

Peter Ho[1], Maria A Quigley[1,2], David Tucker[3], Jennifer J Kurinczuk[1,2]

[1]National Perinatal Epidemiology Unit, Nuffield Department of Population Health, University of Oxford.

[2]National Institute for Health Research (NIHR) Policy Research Unit- Maternal Health and Care, National Perinatal Epidemiology Unit, Nuffield Department of Population Health, University of Oxford.

[3]Congenital Anomaly Register and Information Service (CARIS) in Wales.

Correspondence author: Dr. Peter Ho, National Perinatal Epidemiology Unit, Nuffield Department of Population Health, University of Oxford, Old Road Campus, Headington, Oxford OX3 7LF, UK. Email: Peter.ho@gtc.ox.ac.uk, Peterhosy@hotmail.com Tel: +44 7905497825 Fax: +44 1865 289701.

Word count: 2500 (excluding abstract and references)

Confidential: For Review Only

Abstract

Objectives: To investigate risk factors associated with death of infants with a congenital anomaly in Wales, UK.

Design: A population-based cohort study.

Setting: Data from the Welsh Congenital Anomaly Register and Information Service (CARIS) linked to livebirths and deaths from the Office for National Statistics (ONS).

Patients: All livebirths between 1998 and 2016 with a diagnosis of a congenital anomaly, which was defined as a structural, metabolic, endocrine, or genetic defect, as well as rare disease of hereditary origin.

Main outcome measures: Adjusted odds ratios (aOR) were estimated for socio-demographic, maternal, infant, and intervention factors associated with death in infancy, using logistic regression for all, isolated, multiple and cardiovascular anomalies.

Results: 30,424 livebirths affected by congenital anomalies were identified, including 1,044 infants who died by the age of one year (infant mortality rate: 16.5 per 10,000 livebirths, case fatality: 3.4%, 30.3% of all infant deaths). Risk factors for infant death were non-White vs. White ethnicity (aOR: 2.25; 95% CI: 1.77-2.86); parous vs. nulliparous (aOR: 1.24; 1.08-1.41); smoking during pregnancy vs. non-/ex- smokers (aOR: 1.20; 1.02-1.40); preterm vs. term birth (aOR: 4.38; 3.86-4.98); and female vs. male infants (aOR: 1.28; 1.13-1.46). Infants with a cardiovascular anomaly who received surgery had a lower odds of death than those

Confidential: For Review Only

who did not (aOR: 0.34; 0.15-0.75). Preterm birth was a significant factor for death for all anomalies but the effect of the other characteristics varied according to anomaly group.

Conclusions: Nearly a third of all infant deaths had an associated anomaly. Improving access to prenatal care, smoking cessation advice, optimising care for preterm infants, and surgery may help lower the risk of infant death.

Confidential: For Review Only

Introduction

Congenital anomalies are structural, chromosomal or metabolic abnormalities that occur during intrauterine development[1]; they are the second leading cause of infant death in the UK, accounting for over one-third of all infant deaths[2,3]. To date, population-based studies of risk factors for mortality of infants with congenital anomaly have been limited, with most of the existing studies focusing on a few major anomaly subgroups such as neural tube defects[4,5], and certain cardiovascular and digestive system anomalies[6,7].

Previous studies have shown that socio-demographic, maternal, infant and interventional factors can influence the survival of infants born with specific anomalies. For example, maternal Black ethnicity, preterm birth, cervico-thoracic lesion level of a spina bifida and multiple defects were significantly associated with an increased risk of excess infant deaths in those with neural tube defects[4,5]. Further research on mortality risk factors associated with a wider range of congenital anomalies is needed to inform planning of healthcare and social interventions aimed at reducing infant deaths.

We aimed to investigate risk factors for infant death of infants born with congenital anomalies.

Methods

Study design

A population-based cohort study was conducted using registry data from the Congenital Anomaly Register and Information Service (CARIS) for Wales, linked to births and deaths registration data from the Office for National Statistics (ONS)[3,8] and de-identified for analysis.

Confidential: For Review Only

Study population

The inclusion criteria for this study were: (1) all livebirths between 1998 and 2016 with birthweight ≥500g, gestational age ≥22^{+0} weeks, and a diagnosis of a congenital anomaly reported to CARIS; these infants were followed up for one year after birth. (2) All confirmed and probable cases of congenital anomalies.

Congenital anomalies are defined by CARIS as structural, metabolic, endocrine, or genetic defects, as well as rare diseases of hereditary origin present in the child or fetus at the end of pregnancy, even if not detected until after birth[8]. All congenital anomalies reported to CARIS are coded using the Royal College of Paediatrics and Child Health adaptation of the 10th revision of the International Classification of Diseases and Related Health Problems (ICD-10 RCPCH). Therefore, ICD-10 'Q', 'P35' and 'P37" codes, as well as other non 'Q' ICD-10 codes of congenital anomalies and rare diseases were included in this study.

Categorisation of anomaly

Congenital anomalies were categorised according to the European Surveillance of Congenital Anomalies (EUROCAT) subgroup classification[9]; other rare diseases which are not included in the EUROCAT subgroup classification were categorised according to the ICD chapter headings[10] (Supplementary Table 1).

Infants were considered as having an isolated congenital anomaly (or disease) if a single anomaly (or disease) was diagnosed and reported to CARIS. Infants were considered as having multiple anomalies (or diseases) if more than one anomaly (or diseases) was diagnosed, either within the same body system or involving different body systems. Infants

Confidential: For Review Only

who were diagnosed with a syndrome involving more than one anomaly (or disease) were considered as having multiple anomalies (or diseases).

Variables

To informally assess the validity of data (Supplementary Table 2), CARIS compares data collected by midwives at booking with that collected by fetal cardiologists who also take a history; some discrepancies are noted e.g. maternal smoking and the more complete data are retained by CARIS. Severity of cardiovascular anomalies is based on the EUROCAT classification[29,39].

Statistical analysis

Adjusted odds ratios (aOR) were estimated for socio-demographic, maternal, infant, and intervention factors associated with infant death i.e. death which occurred in the first year after birth. The analysis was performed separately for: infants with any anomaly; those with isolated anomalies; those with multiple anomalies; and those with cardiovascular anomalies.

Infant mortality rate (IMR) was calculated as the number of infant deaths per 10,000 livebirths, where the baby dies before their first birthday. ONS denominator data for IMR was the total number of livebirths in Wales between 1998 and 2016. The risk factors explored were socio-demographic, maternal, infant, and intervention factors (Figure 2); *a priori* factors were identified from the literature, which included maternal ethnicity, infant sex and gestational age at birth[4,5,11,12]. Unadjusted odd ratios (uORs) were estimated in an univariable analysis to identify variables associated with death ($p<0.1$) that were then explored in a multivariable logistic regression model to generate adjusted odds ratios

Confidential: For Review Only

(aORs). Variables were dropped from the multivariable model using a backward stepwise approach if they did not significantly improve the fit of the data (i.e. $p<0.05$ in the likelihood ratio test) with examination of the results as each variable was removed.

For variables including maternal ethnicity, maternal smoking, history of anomalies in previous pregnancy, and infant surgery status, the amount of missing data was high (i.e. 15-41%) and associated with other variables (i.e. not missing completely at random). As it was not possible to exclude that the missingness may also have been at random, multiple imputation was justified and performed for these variables[36] using multiple imputation chained equations (MICE). Sensitivity analyses were conducted using complete case analysis and imputed data to check the robustness of results in the final models. All statistical analyses were performed using Stata 13[13].

Research ethics and statistical disclosure

The de-identified health data for this study was provided by the Secure Anonymised Information Linkage (SAIL) databank; research ethics committee approval was not required for this secondary data analysis study. This study was approved by the SAIL Information Governance Review Panel (IGRP). All statistical disclosure control and checks were strictly followed according to the SAIL Databank and ONS guidelines, to ensure that individual infants were not deductively identifiable.

Patient and public involvement

All proposals to use data within the SAIL Databank are subject to review by an independent Information Governance Review Panel (IGRP) for privacy risk, data governance and public

Confidential: For Review Only

benefit assessment. The IGRP is made up of a range of independent experts as well as members of the public.

Results

A total of 632,945 livebirths occurred to residents in Wales between 1998 and 2016. In this period, 30,424 infants affected by congenital anomalies were identified, of which 20,008 (65.8%) had isolated anomalies and 10,374 (34.1%) had multiple anomalies (Figure 1). There were 1,044 deaths of infants who were affected by either an isolated anomaly or multiple anomalies; this represented an IMR of 16.5 deaths per 10,000 livebirths and case fatality of 3.4% for infants with any anomaly. Infants with an anomaly who died represented almost one-third (30.3%) of all infant deaths (n = 3,443) in this period; about two-thirds (20.7%) of the anomaly related deaths were of infants with multiple anomalies and one-third (9.5%) were of infants with isolated anomalies (Table 1). Among all the isolated anomaly subgroups, cardiovascular anomalies were associated with the largest number of infant deaths (n = 84), representing an IMR of 1.33 deaths per 10,000 livebirths and case fatality of 2.7%.

Table 2 shows the characteristics of the infants with any anomalies who died compared with those who survived infancy. Compared with infants who survived, those who died were more likely to live in the most deprived areas (31% versus 28%), be girls (46% versus 39%), have a low birthweight (51% versus 15%), were born preterm (46% versus 15%), to have not received surgery (45% versus 40%), or had mothers who were of non-White ethnicity (9% versus 3%), multiparous (58% versus 50%), smokers (25% versus 17%), had a multiple

Confidential: For Review Only

pregnancy (11% versus 5%), and a maternal history of an anomaly in a previous pregnancy (11% versus 8%).

Table 3 shows the unadjusted odd ratios for variables associated with infant death ($p < 0.1$) by different anomaly subgroups. Birthweight and gestational age at birth were highly collinear (correlation coefficient $r = 0.75$). Only gestational age was therefore included in the final model, as it has greater clinical utility[14].

Table 4 shows that, for infants with any anomaly, significant increases in the adjusted odds of infant death were found among infants born to mothers who were of non-White ethnicity (aOR 2.25; 95% CI: 1.77-2.86); parous (aOR 1.24; 1.08-1.41); active smokers during pregnancy (aOR 1.20; 1.02-1.40); where the infant was born preterm (aOR 4.38; 3.86-4.98); and was a girl (aOR 1.28; 1.13-1.46). Infants who required surgery in the first year after birth had a lower odds of infant death than those who did not (aOR 0.80; 0.68-0.95). A similar pattern of aORs was seen for isolated anomalies (except that infant sex was not statistically significant) and multiple anomalies (except maternal smoking was not statistically significant). For cardiovascular anomalies, the main risk factors were preterm birth and severity (having a most severe CHD versus less CHD: aOR 229; 90.8-579). Having surgery had a protective effect (aOR 0.34; 0.15-0.75) for infants with a cardiovascular anomaly. These results were not materially different in sensitivity analyses using complete case analysis and imputation (data not shown).

Discussion

This population-based cohort study used linked de-identified data from CARIS and ONS to investigate risk factors contributing to an excess risk of death of infants with congenital

anomalies born to residents in Wales between 1998 and 2016. We found that infants with any congenital anomaly who died before their first birthday were more likely to be girls, be born preterm, not to have received surgery or have mothers who were of non-White ethnicity and smokers. Preterm birth was the strongest risk factor for excess infant deaths across all subgroups of congenital anomalies, but the effects of other factors on excess infant deaths varied according to the anomaly subgroup.

The excess deaths from congenital anomalies in minority ethnic groups is likely to be complex and may be due to an interplay of factors resulting in unequal access to and uptake of antenatal screening and medical and surgical interventions, different attitudes toward congenital anomalies and termination of pregnancy, consanguinity (e.g. a risk factor associated with more lethal anomalies)[30], as well as difference in genetics (e.g. incidence of genotype mutations), culture (e.g. attitude to healthcare and interventions), and behaviour (e.g. maternal smoking) between ethnic groups[4,5,15-18].

The relationship between parity and infant mortality has been well documented although the exact mechanism is not clear; it is thought that biological including the impact of maternal age and sociological factors, as well as factors involving in accessing the health services may play a role[20-22]. Smoking during pregnancy is a well-known risk factor for intrauterine growth restriction, prematurity and fetal death. Previous studies have suggested that placental dysfunction and/or abruption lead to fetal hypoxia due to nicotine-induced vasoconstriction during the perinatal period[23-26]. In addition, smoking has been shown to be associated with an increased risk of sudden infant death syndrome[27]. It is possible that these effects are more serious for infants with an underlying anomaly. However, the literature on smoking in relation to congenital anomalies is not unequivocal.

Confidential: For Review Only

The study of Child Death Outcome Panel data in Bradford showed that smoking in pregnancy appeared protective against infant deaths from congenital anomalies, although the study population was small[37].

Girls with a congenital anomaly had an increased risk of infant death compared to boys with anomalies overall, which is a finding not previous described. However, this is likely to be partially due a male excess of conditions such as pyloric stenosis and potentially hypospadias (not examined in this study), which are sex-specific and rarely lethal (Supplementary Table 3). Infants who are born preterm have an increased risk of co-morbidities and related complications such as intraventricular haemorrhage, chronic lung disease of prematurity and necrotising enterocolitis[28], and hence poorer prognostic outcomes in infancy in general compared with infants born at term. Having a major anomaly also increases the chance of an infant being born preterm[28,29]; potentially, both risk factors may synergistically contribute to the same chain of event leading to infant death and may have a multiplicative effect on the risk ratio[30].

The strong association found between infant mortality risk and severity of cardiovascular anomalies is to be expected. However, classification of disease severity among other anomaly subgroups are less well established, as the aetiology of many congenital anomalies is not known[31]. Advances in diagnostic, surgical and medical interventions have generally improved the survival of infants with congenital anomalies who require surgery; the majority of formally lethal anomalies can now be successfully treated[32]. Particularly, advances in cardiac surgery, imaging, prenatal screening and diagnosis have significantly reduced the risk of death for infants with a congenital heart defect over time[33]. However, death rates remain

Confidential: For Review Only

high for many severe anomalies in spite of medical and surgical interventions, for example, infants with the most severe cardiac conditions continue to experience a significant risk of post-operative cardiovascular sequelae and other complications[34]. We did not find an association between surgery and isolated anomalies, but it is possible that the prognosis of many less severe isolated anomalies is good regardless of surgery compared to multiple anomalies; however, further subgroup analysis of specific anomaly subtypes is needed to confirm this.

This is the first UK population-based cohort study of which we are aware investigating the impact of overall and selected groups of congenital anomalies and rare diseases on infant death. The main strengths include the national study population and robust study design. The study cohort was identified from a high-quality, population-based congenital anomaly registry with an active surveillance system which covers all births in Wales. The multiple source reporting system maximises case finding and thus internal validity[35]. A further strength relates to the broad definition of congenital anomalies, which includes structural and chromosomal defects, as well as rare diseases of congenital origin. Consequently, we were able to generate robust epidemiological findings and assess the full impact of a wide group of congenital anomalies and rare diseases on infant death.

As the occurrence of anomaly-related infant deaths is rare, it was not possible to investigate risk factors for infant mortality for specific anomalies except for cardiovascular anomaly subgroup. Consequently, heterogeneity inevitably exists within anomaly groups in terms of infant mortality risk. In addition, the number of variables explored in this study was restricted by the data available, which is a common limitation of using routinely collected data. The extent of missing data in variables such as maternal ethnicity and smoking is

Confidential: For Review Only

significant, and self-reporting of smoking is not always reliable[38]; therefore, caution should be taken when interpreting these results. In addition, as the Welsh population is predominantly White (>95%), a more nuanced analysis by ethnic subgroups is not possible due to small numbers. Finally, some severe cardiac anomalies are not amenable to surgery, or parents do not wish to put their child through years of major surgical intervention and may instead opt for palliative care, thus potential selection bias cannot be excluded even though surgical intervention has improved over years. While our results were drawn from data from the period between 1998 and 2017, it is important to note that public health interventions such as the smoking cessation programme in Wales from 2007, and the Antenatal Screening Wales in 2003 may have had an impact on the mortality estimates, although assessing the impact of these programmes is outside the scope of this study.

Conclusions

Congenital anomalies are a leading cause of infant death. Socio-demographic, maternal, infant and interventional factors have a significant impact on death in infants with congenital anomalies by likely different potential mechanisms. Improving access to prenatal care, optimising care for preterm infants, smoking cessation advice and surgery may help lower the risk of infant death.

Confidential: For Review Only

What is known about the subject:

- Congenital anomalies are a leading cause of infant death.
- Evidence about the risk factors contributing to the death of infants with congenital anomalies is limited.

What this study adds:

- Preterm birth is the strongest factors significantly associated with excess infant deaths in all anomaly subgroups investigated.
- Girls with an anomaly had an overall increased risk of excess infant death compared to boys with an anomaly, which may be partly due to a male excess in the prevalence of non-lethal anomalies such as pyloric stenosis.
- The effects of maternal ethnicity, maternal smoking, parity, infant sex, anomaly severity and surgery on excess infant case fatality vary according to the anomaly subgroup.

Confidential: For Review Only

Data sharing statement

The de-identified health data for this study was provided by the Secure Anonymised Information Linkage (SAIL) databank.

Acknowledgements

We thank the SAIL team for providing the datasets and their assistance with data linkage for this study, and the CARIS team for their support with the CARIS data.

Funding

This study is part-funded by the National Institute for Health Research (NIHR) Policy Research Programme, conducted through the NIHR Policy Research Unit in Maternal Health and Care, 108/0001. The views expressed are those of the author(s) and not necessarily those of the NIHR or the Department of Health and Social Care.

Peter Ho is funded by a Clarendon – Green Templeton College – Nuffield Department of Population Health Scholarship, University of Oxford.

Competing interests None declared.

Contributorship statement:

PH contributed to the design of the study, acquisition, analysis, interpretation of data, drafting and revising it critically, approved the final version to be published, and agrees to be accountable for all aspects of the work in ensuring that questions related to the accuracy or integrity of any part of the work are appropriately investigated and resolved.

MQ and JK contributed to the design of the study, acquisition, analysis, and interpretation of data for the work; supervised the project and revised it critically; approved the final version to be published; and agree to be accountable for all aspects of the work in ensuring that questions related to the accuracy or integrity of any part of the work are appropriately investigated and resolved.

DT contributed to the acquisition, interpretation of data for the study, revised it critically, approved the final version to be published, and agrees to be accountable for all aspects of the work in ensuring that questions related to the accuracy or integrity of any part of the work are appropriately investigated and resolved.

https://mc.manuscriptcentral.com/bmjpo

Confidential: For Review Only

Table 1: Infant mortality rate by congenital anomaly subgroup in Wales (1998-2016 birth cohort).

Anomaly subgroup[2]	Livebirths N = 632,945[1], (%)	Deaths (%) N = 3,443[2] (%)	Case fatality	IMR per 10,000 livebirths
All anomalies[3]	30,424 (4.8)	1,044 (30.3)	3.4%	16.5
All isolated anomalies	20,008 (3.2)	327 (9.5)	1.6%	5.17
All multiple anomalies	10,374 (1.6)	714 (20.7)	6.9%	11.3
Isolated cardiovascular anomalies	3,149 (0.5)	84 (2.4)	2.7%	1.33

[1]Total number of livebirths in Wales between 1998 and 2016 (ONS data) as the denominator. [2]Total number of deaths in Wales between 1998 and 2016 (ONS data) as the denominator. [3]The number of isolated and multiple anomalies does not sum to all anomalies due to coding issues of <0.1% of cases.

Confidential: For Review Only

Table 2: Descriptive characteristics of infants with any anomaly who died and those who survived in the first year after birth.

	Died in infancy, n (%)	Survival infancy, n (%)
Socio-demographic factor		
Townsend quintile		
1 (least deprived)	122 (12)	4,564 (16)
2	160 (15)	4,594 (16)
3	207 (20)	5,361 (19)
4	225 (22)	5,861 (21)
5 (most deprived)	327 (31)	7,886 (28)
Maternal ethnicity		
White	715 (69)	15,807 (56)
Other	89 (9)	902 (3)
Not known/ missing	240 (23)	11,646 (41)
Maternal age at birth, years *(n = 29,400)*		
Mean +/- SD (year)	*28.3 +/- 6.5*	*28.2 +/- 6.2*
≤ 24	318 (31)	8,514 (30)
25 – 29	288 (28)	7,835 (28)
30 – 34	239 (23)	7,129 (25)
≥ 35	199 (19)	4,849 (17)
Maternal factors		
Parity		
Nulliparous	385 (37)	11,115 (39)
≥ 1	606 (58)	14,116 (50)
Multiple pregnancy		
Yes	114 (11)	1,334 (5)
No	930 (89)	27,022 (95)
Maternal smoking		
Smoker	261 (25)	4,712 (17)
Non/ Ex smoker	541 (52)	12,110 (42)
Not known / missing	242 (23)	11,534 (41)
Anomalies in previous pregnancies		
Yes	117 (11)	2,257 (8)
No	724 (69)	16,153 (57)
Not known / missing	203 (19)	9,946 (35)
Infant factors		
Infant sex		
Male	566 (54)	17,810 (61)
Female	474 (46)	11,550 (39)
Birthweight, grams		
Median (IQR)	*2475 (1650-3150)*	*3250 (2780-3650)*
< 2500 (low birthweight)	529 (51)	4,505 (15)
≥ 2500	510 (49)	23,835 (81)
Gestational age, week		
Median (IQR)	*37 (32-39)*	*39 (38-40)*
$< 37^{+0}$ (preterm)	480 (46)	4,510 (15)
$\geq 37^{+0}$ (term)	562 (54)	23,935 (82)
Intervention factors		
Surgery		
Performed (or expected) in the first year after birth	216 (21)	6,405 (22)
Not performed or required in the first year after birth[2]	465 (45)	11,833 (40)
Not known/ missing	363 (35)	11,142 (38)

Category with small number is not shown due to risk of statistical disclosure. [2]Including a minority of conditions which were too severe for surgery. Note: Confirmed and probable cases were included in the analysis.

Confidential: For Review Only

Table 3: Unadjusted odds ratios for infant mortality by anomaly subgroup.

Factors	All anomalies	Isolated anomalies	Multiple anomalies	Cardiovascular anomalies
Townsend quintile				
1 (least deprived)	1 (reference)	1 (reference)	1 (reference)	1 (reference)
2	1.30 (1.03-1.65)	1.30 (0.86-1.95)	1.29 (0.96-1.73)	1.61 (0.76-3.41)
3	1.44 (1.15-1.81)	1.27 (0.85-1.89)	1.48 (1.12-1.96)	1.25 (0.59-2.66)
4	1.44 (1.15-1.80)	1.35 (0.92-2.00)	1.37 (1.04-1.80)	0.91 (0.41-2.01)
5 (most deprived)	1.55 (1.26-1.92)	1.46 (1.02-2.11)	1.56 (1.20-2.02)	1.33 (0.67-2.66)
Maternal ethnicity[1]				
White	1 (reference)	1 (reference)	1 (reference)	1 (reference)
Other	2.18 (1.73-2.75)	1.77 (1.11-2.82)	2.34 (1.78-3.08)	1.00 (0.31-3.26)
Not known/ missing	0.46 (0.39-0.53)	0.59 (0.47-0.75)	0.56 (0.46-0.68)	0.71 (0.44-1.13)
Maternal age at birth, years				
25-29	1 (reference)	1 (reference)	1 (reference)	1 (reference)
≤ 24	1.02 (0.86-1.20)	1.04 (0.79-1.37)	0.97 (0.79-1.19)	1.12 (0.64-1.96)
30-34	0.91 (0.77-1.09)	0.77 (0.56-1.05)	0.96 (0.78-1.19)	0.77 (0.41-1.45)
≥ 35	1.12 (0.93-1.34)	0.92 (0.65-1.28)	1.12 (0.90-1.41)	1.14 (0.60-2.18)
Parity				
Nulliparous	1 (reference)	1 (reference)	1 (reference)	1 (reference)
≥ 1	1.24 (1.09-1.41)	1.48 (1.16-1.89)	1.18 (1.01-1.39)	1.16 (0.72-1.85)
Not known/ missing	0.49 (0.37-0.65)	0.99 (0.67-1.46)	0.37 (0.23-0.61)	1.16 (0.52-2.56)
Multiple pregnancy				
No	1 (reference)	1 (reference)	1 (reference)	1 (reference)
Yes	2.48 (2.03-3.04)	2.57 (1.80-3.65)	2.35 (1.83-3.04)	1.40 (0.64-3.08)
Maternal smoking				
Non/ Ex smoker	1 (reference)	1 (reference)	1 (reference)	1 (reference)
Smoker	1.24 (1.07-1.44)	1.52 (1.15-2.01)	1.11 (0.92-1.33)	1.91 (1.12-3.25)
Not known / missing	0.47 (0.40-0.55)	0.66 (0.52-0.85)	0.57 (0.46-0.70)	0.85 (0.51-1.43)
Anomalies in previous pregnancies				
No	1 (reference)	1 (reference)	1 (reference)	1 (reference)
Yes	1.16 (0.95-1.41)	1.24 (0.86-1.79)	1.25 (0.98-1.59)	0.56 (0.20-1.56)
Not known / missing	0.46 (0.39-0.53)	0.70 (0.55-0.89)	0.53 (0.42-0.66)	0.96 (0.60-1.53)
Infant sex[1]				
Male	1 (reference)	1 (reference)	1 (reference)	1 (reference)
Female	1.29 (1.14-1.46)	1.08 (0.86-1.35)	1.33 (1.14-1.55)	0.79 (0.51-1.22)
Birthweight, grams[2]				
≥ 2500	1 (reference)	1 (reference)	1 (reference)	1 (reference)
< 2500 (low birthweight)	5.49 (4.84-6.22)	5.32 (4.26-6.64)	4.54 (3.88-5.30)	3.34 (2.13-5.23)
Gestational age at birth[1,2]				
$\geq 37^{+0}$ (term)	1 (reference)	1 (reference)	1 (reference)	1 (reference)
$< 37^{+0}$ (preterm)	4.53 (4.00-5.14)	4.72 (3.78-5.90)	3.84 (3.29-4.49)	2.76 (1.75-4.35)
CVS anomaly severity	n/a	n/a	n/a	
Less severity				1 (reference)
Moderate severity				10.1 (4.9-21.1)
Most severity				90.7 (43.4-189)

Confidential: For Review Only

Table 3 (continue): Unadjusted odds ratios for infant mortality by anomaly subgroup.

Factors	All anomalies	Isolated anomalies	Multiple anomalies	Cardiovascular anomalies
Surgery				
Not performed or required in the first year after birth[5]	1 (reference)	1 (reference)	1 (reference)	1 (reference)
Performed (or expected) in the first year after birth	0.86 (0.73-1.01)	0.80 (0.58-1.10)	0.68 (0.56-0.82)	2.93 (1.66-5.17)
Not known/ missing	0.83 (0.72-0.95)	0.92 (0.73-1.17)	0.76 (0.64-0.91)	0.75 (0.44-1.26)

[1]a priori variable; [2]Gestational age at birth and birthweight are strongly correlated (r ≥ 0.7). Note: Statistical significance at p < 0.1; NS = non-significant.

Confidential: For Review Only

Table 4: Adjusted odds ratios for factors associated with infant death by anomaly subgroup.

Factors	All anomalies	Isolated anomalies	Multiple anomalies	Cardiovascular anomalies
Ethnicity<br>Other vs. White	2.25 (1.77-2.86)	1.87 (1.16-3.02)	2.38 (1.79-3.16)	1.08 (0.29-4.04)
Parity<br>≥ 1 vs. Nulliparous	1.24 (1.08-1.41)	1.48 (1.16-1.89)	1.19 (1.02-1.40)	
Maternal smoking<br>Smoker vs. Non/Ex smoker	1.20 (1.02-1.40)	1.44 (1.07-1.92)		
Infant sex<br>Female vs. Male	1.28 (1.13-1.46)	1.06 (0.85-1.33)	1.37 (1.17-1.61)	1.26 (0.78-2.06)
Gestational age at birth<br>Preterm vs. Term	4.38 (3.86-4.98)	4.53 (3.62-5.67)	3.86 (3.30-4.53)	3.67 (2.19-6.16)
CHD severity<br>Moderate vs. Less<br>Most vs. Less	n/a	n/a	n/a	18.4 (8.10-41.7)<br>229 (90.8-579)
Surgery<br>Yes vs. No	0.80 (0.68-0.95)		0.71 (0.58-0.86)	0.34 (0.15-0.75)

Note: Statistical significance at $p < 0.05$; Adjusted odd ratios with 95% confidence intervals are shown. Empty cell represents variable that was not included in the multivariable analysis. n/a = not applicable.

Confidential: For Review Only

References

1. European Surveillance of Congenital Anomalies (EUROCAT) 2019, *EUROCAT Network Overview: Congenital anomalies and public health,* EUROCAT, viewed 26 August 2019, <https://eu-rd-platform.jrc.ec.europa.eu/eurocat/eurocat-network/eurocat-network-overview_en#inline-nav-2>.

2. Public Health England (PHE) 2019, National Congenital Anomaly and Rare Disease Registration Service Congenital Anomaly Statistics 2017, PHE, viewed 8 September 2018, <https://assets.publishing.service.gov.uk/government/uploads/system/uploads/attachment_data/file/821449/NCARDRS_congenital_anomaly_stats_2017.pdf>.

3. Office for National Statistics (ONS) 2015, *Statistical bulletin: Birth Summary Tables, England and Wales: 2014,* ONS, viewed 8 September 2019, <https://www.ons.gov.uk/peoplepopulationandcommunity/birthsdeathsandmarriages/livebirths/bulletins/birthsummarytablesenglandandwales/2015-07-15>.

4. Bol, K.A., Collins, J.S. and Kirby, R.S., 2006. Survival of infants with neural tube defects in the presence of folic acid fortification. *Pediatrics*, *117*(3), pp.803-813.

5. Shin, M., Kucik, J.E., Siffel, C., Lu, C., Shaw, G.M., Canfield, M.A. and Correa, A., 2012. Improved survival among children with spina bifida in the United States. *The Journal of pediatrics*, *161*(6), pp.1132-1137.

6. Best, K.E. and Rankin, J., 2016. Long-term survival of individuals born with congenital heart disease: a systematic review and meta-analysis. *Journal of the American Heart Association*, *5*(6), p.e002846.

7. Clark, R.H., Walker, M.W. and Gauderer, M.W.L., 2011. Factors associated with mortality in neonates with gastroschisis. *European Journal of Pediatric Surgery*, *21*(01), pp.21-24.

8. Congenital Anomaly Register and Information Service (CARIS) 2018, *Introduction. Congenital Anomaly Register and information Service*, CARIS, viewed 8 March 2019, <http://www.caris.wales.nhs.uk/introduction>.

9. European Surveillance of Congenital Anomalies (EUROCAT) 2013, EUROCAT Guide 1.4: Instruction for the registration of congenital anomalies. EUROCAT Central Registry, University of Ulster. Last update version 28/12/2018, viewed 25 August 2019, <https://eu-rd-platform.jrc.ec.europa.eu/sites/default/files/Full_Guide_1_4_version_28_DEC2018.pdf>.

Confidential: For Review Only

10. World Health Organization (WHO), 2004. ICD-10: international statistical classification of diseases and related health problems: tenth revision.

11. Melamed, N., Yogev, Y. and Glezerman, M., 2010. Fetal gender and pregnancy outcome. *The Journal of Maternal-Fetal & Neonatal Medicine*, *23*(4), pp.338-344.

12. Lisi, A., Botto, L.D., Rittler, M., Castilla, E., Bianchi, F., Botting, B., De Walle, H., Erickson, J.D., Gatt, M., De Vigan, C. and Irgens, L., 2005. Sex and congenital malformations: an international perspective. *American Journal of Medical Genetics Part A*, *134*(1), pp.49-57.

13. StataCorp. Stata multiple-imputation reference manual. College Station, TX: Stata Press, 2013: 48.

14. Ruangtrakool, R., Laohapensang, M., Sathornkich, C. and Talalak, P., 2001. Necrotizing enterocolitis: a comparison between full-term and pre-term neonates. *Journal of the Medical Association of Thailand= Chotmaihet thangphaet*, *84*(3), p.323.

15. Weightman, A.L., Morgan, H.E., Shepherd, M.A., Kitcher, H., Roberts, C. and Dunstan, F.D., 2012. Social inequality and infant health in the UK: systematic review and meta-analyses. *BMJ open*, *2*(3), p.e000964.

16. Dominguez, T.P., Dunkel-Schetter, C., Glynn, L.M., Hobel, C. and Sandman, C.A., 2008. Racial differences in birth outcomes: the role of general, pregnancy, and racism stress. *Health psychology*, *27*(2), p.194.

17. Gray, R., Headley J., Oakley L., Kurinczuk JJ., Brocklehurst P., Hollowell J. Inequalities in infant mortality project briefing paper 3. Towards an understanding of variations in infant mortality rates between different ethnic groups. Oxford: National Perinatal Epidemiology Unit, 2009.

18. Botto, L.D. and Yang, Q., 2000. 5, 10-Methylenetetrahydrofolate reductase gene variants and congenital anomalies: a HuGE review. *American journal of epidemiology*, *151*(9), pp.862-877.

19. Sonneveldt, E., Plosky, W.D. and Stover, J., 2013. Linking high parity and maternal and child mortality: what is the impact of lower health services coverage among higher order births?. *BMC public health*, *13*(3), p.S7.

20. Hook, E.B., 1985. The impact of aneuploidy upon public health: mortality and morbidity associated with human chromosome abnormalities. In *Aneuploidy* (pp. 7-33). Springer, Boston, MA.

Confidential: For Review Only

21. Cuckle, H.S., 2005. Primary prevention of Down's syndrome. *International journal of medical sciences*, *2*(3), p.93.

22. Hobcraft, J., McDonald, J.W. and Rutstein, S., 1983. Child-spacing effects on infant and early child mortality. *Population Index*, pp.585-618.

23. Rogers JM. Tobacco and pregnancy. Reprod Toxicol. 2009;28:152–160.

24. Quinton, A.E., Cook, C.M. and Peek, M.J., 2008. The relationship between cigarette smoking, endothelial function and intrauterine growth restriction in human pregnancy. *BJOG: An International Journal of Obstetrics & Gynaecology*, *115*(6), pp.780-784.

25. Tikkanen, M., 2011. Placental abruption: epidemiology, risk factors and consequences. *Acta obstetricia et gynecologica Scandinavica*, *90*(2), pp.140-149.

26. Andres, R.L. and Day, M.C., 2000, August. Perinatal complications associated with maternal tobacco use. In *Seminars in Neonatology* (Vol. 5, No. 3, pp. 231-241). Elsevier.

27. Mitchell, E.A., 1995. Smoking: the next major and modifiable risk factor. *Sudden infant death syndrome: New trends in the nineties*, pp.114-118.

28. Butler, A.S. and Behrman, R.E. eds., 2007. *Preterm birth: causes, consequences, and prevention*. National academies press.

29. Khoshnood, B., Lelong, N., Houyel, L., Thieulin, A.C., Jouannic, J.M., Magnier, S., Delezoide, A.L., Magny, J.F., Rambaud, C., Bonnet, D. and Goffinet, F., 2012. Prevalence, timing of diagnosis and mortality of newborns with congenital heart defects: a population-based study. *Heart*, *98*(22), pp.1667-1673.

30. Shawky, R.M., Elsayed, S.M., Zaki, M.E., El-Din, S.M.N. and Kamal, F.M., 2013. Consanguinity and its relevance to clinical genetics. *Egyptian Journal of Medical Human Genetics*, *14*(2).

31. World Health Organization (WHO) 2020, *Congenital anomalies: causes,* WHO, viewed 29 April 2020, <https://www.who.int/health-topics/congenital-anomalies#tab=tab_2>.

32. Farmer, D., Sitkin, N., Lofberg, K., Donkor, P. and Ozgediz, D., 2015. Surgical interventions for congenital anomalies.

Confidential: For Review Only

33. Holst, K.A., Said, S.M., Nelson, T.J., Cannon, B.C. and Dearani, J.A., 2017. Current interventional and surgical management of congenital heart disease: specific focus on valvular disease and cardiac arrhythmias. *Circulation research*, *120*(6), pp.1027-1044.

34. McCracken, C., Spector, L.G., Menk, J.S., Knight, J.H., Vinocur, J.M., Thomas, A.S., Oster, M.E., St Louis, J.D., Moller, J.H. and Kochilas, L., 2018. Mortality Following Pediatric Congenital Heart Surgery: An Analysis of the Causes of Death Derived From the National Death Index. *Journal of the American Heart Association*, *7*(22), p.e010624.

35. Congenital Anomaly Register and Information Service (CARIS) 2020a, *Introduction: Reporting to CARIS*, CARIS, viewed 12 March 2020, <http://www.caris.wales.nhs.uk/reporting-to-caris>.

36. Hughes, R.A., Heron, J., Sterne, J.A. and Tilling, K., 2019. Accounting for missing data in statistical analyses: multiple imputation is not always the answer. *International journal of epidemiology*, *48*(4), pp.1294-1304.

37. Firth, C., Petherick, E. and Oddie, S.J., 2018. Infant deaths from congenital anomalies: novel use of Child Death Overview Panel data. *Archives of disease in childhood*, *103*(11), pp.1027-1032.

38. Shipton, D., Tappin, D.M., Vadiveloo, T., Crossley, J.A., Aitken, D.A. and Chalmers, J., 2009. Reliability of self reported smoking status by pregnant women for estimating smoking prevalence: a retrospective, cross sectional study. *Bmj*, *339*.

39. European Surveillance of Congenital Anomalies (EUROCAT) 2020a, *Special Report on Congenital Heart Defects.* EUROCAT Central Registry, University of Ulster, EUROCAT, viewed 29 April 2020, <http://www.eurocat-network.eu/content/Special-Report-CHD.pdf>.

40. Khoshnood, B., Lelong, N., Houyel, L., Thieulin, A.C., Jouannic, J.M., Magnier, S., Delezoide, A.L., Magny, J.F., Rambaud, C., Bonnet, D. and Goffinet, F., 2012. Prevalence, timing of diagnosis and mortality of newborns with congenital heart defects: a population-based study. *Heart*, *98*(22), pp.1667-1673.

Confidential: For Review Only

Figure 1: Anomaly status and categories included in this study; births in Wales 1998 to 2016.

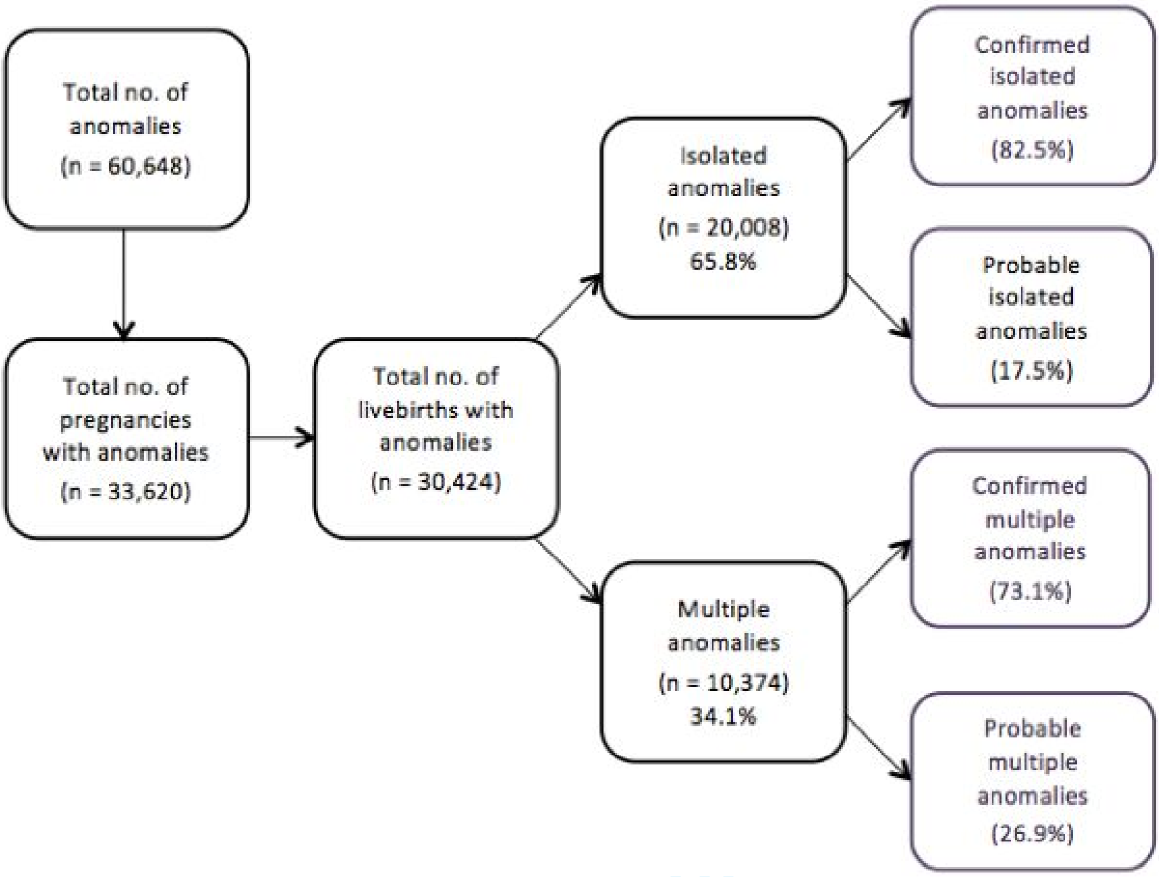

Note: The number of isolated and multiple anomalies does not sum to all anomalies due to coding issues of <0.1% of cases.

For peer review only

Figure 2: Chronological pathway between socio-demographic, maternal, infant's and intervention factors, in relation to infant death.

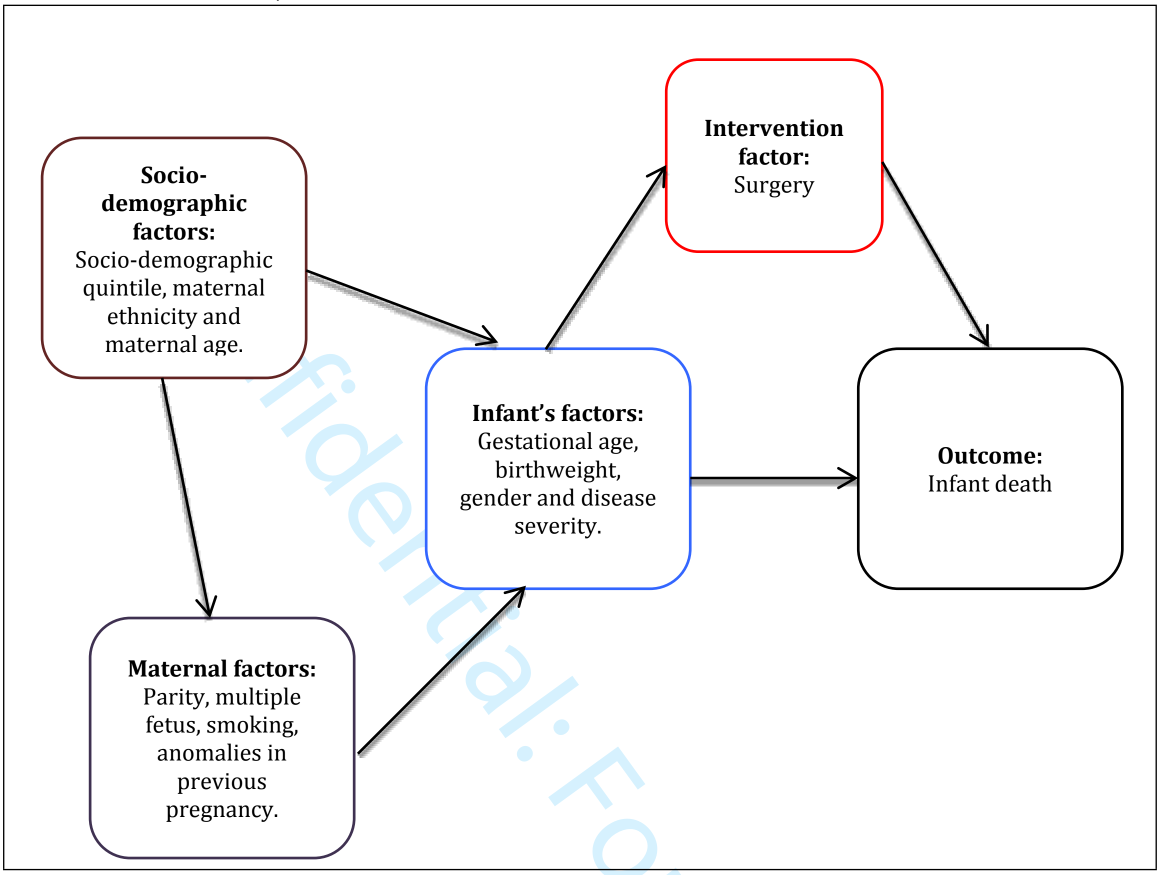

Confidential: For Review Only

Supplementary Table 1: Inclusion of anomaly subgroups.

Subgroup	Classification of anomaly included
All anomalies; all isolated anomalies; and all multiple anomalies	• Nervous system;<br>• Eye, ear, face and neck;<br>• Cardiovascular system;<br>• Respiratory and clefts;<br>• Abdominal wall defects and diaphragmatic hernia;<br>• Upper and lower gastrointestinal;<br>• Genitourinary;<br>• Limb and skeletal;<br>• Blood disorders;<br>• Endocrine and Metabolic;<br>• Neoplasm;<br>• Syndromes and congenital malformation syndromes;<br>• Maternal Infection leading to malformations.
Cardiovascular anomalies	• Congenital heart defects and diseases of the circulatory system.

Confidential: For Review Only

Supplementary Table 2: Description of included variables in the analyses.

Variable	Categorisation
Townsend (as area-based deprivation)	1 (least deprived)[1], 2, 3, 4, 5 (most deprived), and not known/missing.
Maternal ethnicity	White[2], Other (Chinese, Indian, Bangladeshi, Pakistani, Other Asian, Black Caribbean, Black African, Other Black, Mixed, Other), and not known/missing.
Maternal age at birth	≤24, 25-29[2], 30-34, ≥35 years, and not known/missing.
Parity	Nulliparous[2], ≥1, and not known/missing.
Multiple pregnancies	Yes, No[2], and not known/missing.
Maternal smoking	Smoker, Non/ Ex-smoker[2], and not known/missing.
Anomaly in previous pregnancies	Yes, No[2], and not known/missing.
Infant sex	Male, Female[2] and not known/missing.
Infant's birthweight	<2500 (low birthweight), ≥2500[2] g and not known/missing.
Infant's gestational age at birth[2]	$<37^{+0}$ (preterm), $\geq37^{+0}$ [(1)], and not known/missing.
Disease severity (for CHD only)[3]	Less severity[1], moderate severity, most severity, and not known/missing.
Surgery	Performed (or expected) in the first year after birth, not performed or required in the first year after birth[1], and not known/missing.

[1]Reference group. [2]Significant departure of linearity was shown in the effect of gestational age in weeks on infant mortality.[3]Congenital heart defects (CHD) severity category was based on criteria used by Khoshnood et al. (2012): CHD less severe = Ventricular septal defect (VSD), Atrial septal defect (ASD), Pulmonary valve stenosis. CHD moderate severe = Common arterial truncus, Transposition of great vessels, Atrio-ventricular septal defect (AVSD), Tetralogy of Fallot, Pulmonary valve atresia, Aortic valve atresia/ stenosis, Coarctation of aorta, total anomalous pulmonary venous return. CHD most severe = Single ventricle, Tricuspid atresia and stenosis, Ebstein's anomaly, Hypoplastic left heart and Hypoplastic right heart.

Confidential: For Review Only

Supplementary Table 3: Unadjusted odd ratios for sex and mortality stratified by congenital anomaly subgroups.

	Female		Male		OR (95% CI) for female vs. male
**Anomaly subgroup[1,2]**	**Total**	**Died %**	**Total**	**Died (%)**	
Nervous system	734	12.26	847	11.22	1.09 (0.83-1.43)
Eye, ear, face and neck	1,826	4.50	2.032	4.72	0.95 (0.71-1.28)
Cardiovascular	4,580	7.47	4,837	6.86	1.09 (0.94-1.26)
Respiratory and clefts	811	7.64	1,076	7.71	0.99 (0.72-1.36)
Abdominal wall defects and diaphragmatic hernia	272	10.29	378	11.38	0.89 (0.57-1.40)
Gastrointestinal (GI)	961	5.41	2,377	3.83	1.41 (1.01-1.97)
Some subtypes of GI					
Anomalies of the tongue and mouth, oesophageal atresia or stenosis and tracheo-oesophageal fistula	124	7.26	208	6.25	1.16 (0.51-2.64)
Pyloric stenosis	185	2.70	1081	0.74	3.65 (1.21-11.04)
Atresia/ stenosis of duodenum and small intestine	163	3.68	196	8.16	0.45 (0.18-1.13)
Hirschsprung's disease and other malformations of intestine	230	4.35	276	3.26	1.33 (0.55-3.23)
Genitourinary	1,658	1.57	7,489	1.26	1.25 (0.81-1.92)
Limb and skeletal	3,150	1.97	2,875	3.76	0.52 (0.39-0.71)
Blood disorders	142	9.86	253	4.35	2.27 (1.06-4.86)
Anaemias, coagulation defects, purpura and other haemorrhagic conditions	106	4.72	a	-	-
Diseases of blood and blood-forming organs/ disorders involving the immune mechanism	36	25.00	46	15.22	1.64 (0.68-3.99)
Endocrine and metabolic	770	4.81	810	4.44	1.08 (0.69-1.69)
Neoplasm	400	5.25	358	3.35	1.57 (0.78-3.14)
Syndrome	3,087	21.61	3,691	14.90	1.45 (1.31-1.61)
Some subtypes of syndrome					
DiGeorge syndrome	119	7.56	145	11.03	0.69 (0.31-1.50)
Down syndrome	723	13.83	877	11.86	1.17 (0.90-1.51)
Edward syndrome	201	85.07	94	84.04	1.01 (0.91-1.13)
Patau syndrome	80	87.50	39	87.18	1.00 (0.87-1.17)
Total	19,535	8.00	28,738	5.80	1.38 (1.29-1.47)

[1]Anomalies within each anomaly subgroup can be isolated anomalies or part of multiple anomalies. [2]Observations in categories may not add up to the total, as some of the observations are not shown due to small number or risk of statistical disclosure. a = n < 5 or risk of statistical disclosure are not shown.

Confidential: For Review Only

Confidential: For Review Only

BMJ Paediatrics Open

RISK FACTORS FOR DEATH IN WELSH INFANTS WITH A CONGENITAL ANOMALY.

Journal:	*BMJ Paediatrics Open*
Manuscript ID	bmjpo-2021-001205.R1
Article Type:	Original research
Date Submitted by the Author:	14-Nov-2021
Complete List of Authors:	Ho, Peter ; University of Oxford, National Perinatal Epidemiology Unit, Nuffield Department of Population Health<br>Quigley, Maria; University of Oxford, National Perinatal Epidemiology Unit, Nuffield Department of Population Health; University of Oxford, National Institute for Health Research (NIHR) Policy Research Unit-Maternal Health and Care, National Perinatal Epidemiology Unit, Nuffield Department of Population Health.<br>Tucker, David; Public Health Wales, Congenital Anomaly Register and Information Service for Wales<br>Kurinczuk, Jenny; University of Oxford, National Perinatal Epidemiology Unit, Nuffield Department of Population Health; University of Oxford, National Institute for Health Research (NIHR) Policy Research Unit-Maternal Health and Care, National Perinatal Epidemiology Unit, Nuffield Department of Population Health.
Keywords:	Epidemiology, Mortality, Neonatology

SCHOLARONE™
Manuscripts

I, the Submitting Author has the right to grant and does grant on behalf of all authors of the Work (as defined in the below author licence), an exclusive licence and/or a non-exclusive licence for contributions from authors who are: i) UK Crown employees; ii) where BMJ has agreed a CC-BY licence shall apply, and/or iii) in accordance with the terms applicable for US Federal Government officers or employees acting as part of their official duties; on a worldwide, perpetual, irrevocable, royalty-free basis to BMJ Publishing Group Ltd ("BMJ") its licensees and where the relevant Journal is co-owned by BMJ to the co-owners of the Journal, to publish the Work in this journal and any other BMJ products and to exploit all rights, as set out in our licence.

The Submitting Author accepts and understands that any supply made under these terms is made by BMJ to the Submitting Author unless you are acting as an employee on behalf of your employer or a postgraduate student of an affiliated institution which is paying any applicable article publishing charge ("APC") for Open Access articles. Where the Submitting Author wishes to make the Work available on an Open Access basis (and intends to pay the relevant APC), the terms of reuse of such Open Access shall be governed by a Creative Commons licence – details of these licences and which Creative Commons licence will apply to this Work are set out in our licence referred to above.

Other than as permitted in any relevant BMJ Author's Self Archiving Policies, I confirm this Work has not been accepted for publication elsewhere, is not being considered for publication elsewhere and does not duplicate material already published. I confirm all authors consent to publication of this Work and authorise the granting of this licence.

Confidential: For Review Only

RISK FACTORS FOR DEATH IN WELSH INFANTS WITH A CONGENITAL ANOMALY.

Peter Ho[1], Maria A Quigley[1,2], David Tucker[3], Jennifer J Kurinczuk[1,2]

[1]National Perinatal Epidemiology Unit, Nuffield Department of Population Health, University of Oxford.

[2]National Institute for Health Research (NIHR) Policy Research Unit- Maternal Health and Care, National Perinatal Epidemiology Unit, Nuffield Department of Population Health, University of Oxford.

[3]Congenital Anomaly Register and Information Service (CARIS) in Wales.

Correspondence author: Dr. Peter Ho, National Perinatal Epidemiology Unit, Nuffield Department of Population Health, University of Oxford, Old Road Campus, Headington, Oxford OX3 7LF, UK. Email: Peterhosy@hotmail.com Tel: +44 7905497825 Fax: +44 1865 289701.

Word count: 2500 (excluding abstract and references)

Confidential: For Review Only

Abstract

Objectives: To investigate risk factors associated with death of infants with a congenital anomaly in Wales, UK.

Design: A population-based cohort study.

Setting: Data from the Welsh Congenital Anomaly Register and Information Service (CARIS) linked to livebirths and deaths from the Office for National Statistics (ONS).

Patients: All livebirths between 1998 and 2016 with a diagnosis of a congenital anomaly, which was defined as a structural, metabolic, endocrine, or genetic defect, as well as rare disease of hereditary origin.

Main outcome measures: Adjusted odds ratios (aOR) were estimated for socio-demographic, maternal, infant, and intervention factors associated with death in infancy, using logistic regression for all, isolated, multiple and cardiovascular anomalies.

Results: 30,424 livebirths affected by congenital anomalies were identified, including 1,044 infants who died by the age of one year (infant mortality rate: 16.5 per 10,000 livebirths, case fatality: 3.4%, 30.3% of all infant deaths). Risk factors for infant death were non-White vs. White ethnicity (aOR: 2.25; 95% CI: 1.77-2.86); parous vs. nulliparous (aOR: 1.24; 1.08-1.41); smoking during pregnancy vs. non-/ex- smokers (aOR: 1.20; 1.02-1.40); preterm vs. term birth (aOR: 4.38; 3.86-4.98); female vs. male infants (aOR: 1.28; 1.13-1.46) and the earlier years of the birth cohort (aOR 0.96; 0.95-0.98 per yearly increase). Infants with a cardiovascular anomaly who received surgery had a lower odds of death than those who did

Confidential: For Review Only

not (aOR: 0.34; 0.15-0.75). Preterm birth was a significant factor for death for all anomalies but the effect of the other characteristics varied according to anomaly group.

Conclusions: Nearly a third of all infant deaths had an associated anomaly. Improving access to prenatal care, smoking cessation advice, optimising care for preterm infants, and surgery may help lower the risk of infant death.

Confidential: For Review Only

Introduction

Congenital anomalies are structural, chromosomal or metabolic abnormalities that occur during intrauterine development [1]; they are the second leading cause of infant death in the UK, accounting for over one-third of all infant deaths [2-3]. To date, population-based studies of risk factors for mortality of infants with congenital anomaly have been limited, with most of the existing studies focusing on a few major anomaly subgroups such as neural tube defects [4-5], and certain cardiovascular and digestive system anomalies [6-7].

Previous studies have shown that socio-demographic, maternal, infant and interventional factors can influence the survival of infants born with specific anomalies. For example, maternal Black ethnicity, preterm birth, cervico-thoracic lesion level of a spina bifida and multiple defects were significantly associated with an increased risk of excess infant deaths in those with neural tube defects [4-5]. Further research on mortality risk factors associated with a wider range of congenital anomalies is needed to inform planning of healthcare and social interventions aimed at reducing infant deaths.

We aimed to investigate risk factors for infant death of infants born with congenital anomalies.

Methods

Study design

A population-based cohort study was conducted using registry data from the Congenital Anomaly Register and Information Service (CARIS) for Wales, linked to births and deaths

Confidential: For Review Only

registration data from the Office for National Statistics (ONS) [3, 8] and de-identified for analysis.

Study population

The inclusion criteria for this study were all livebirths between 1998 and 2016 with birthweight ≥500g, gestational age ≥22^{+0} weeks, and a confirmed or probable diagnosis of a congenital anomaly reported to CARIS; these infants were followed up for one year after birth.

Congenital anomalies are defined by CARIS as structural, metabolic, endocrine, or genetic defects, as well as rare diseases of hereditary origin present in the child or fetus at the end of pregnancy, even if not detected until after birth [8]. All congenital anomalies reported to CARIS are coded using the Royal College of Paediatrics and Child Health adaptation of the 10th revision of the International Classification of Diseases and Related Health Problems (ICD-10 RCPCH). Therefore, ICD-10 'Q', 'P35' and 'P37" codes, as well as other non 'Q' ICD-10 codes of congenital anomalies and rare diseases were included in this study.

Categorisation of anomaly

Congenital anomalies were categorised according to the European Surveillance of Congenital Anomalies (EUROCAT) subgroup classification [9]; other rare diseases which are not included in the EUROCAT subgroup classification were categorised according to the ICD chapter headings [10] (Supplementary Table 1).

Infants were considered as having an isolated congenital anomaly (or disease) if a single anomaly (or disease) was diagnosed and reported to CARIS. Infants were considered as

Confidential: For Review Only

having multiple anomalies (or diseases) if more than one anomaly (or diseases) was diagnosed, either within the same body system or involving different body systems, for example, an infant was diagnosed with a spina bifida and a ventricular septal defect. Infants who were diagnosed with a syndrome involving more than one anomaly (or disease) were considered as having multiple anomalies (or diseases), for example, an infant was diagnosed with a Down syndrome and a congenital heart defect.

Variables

To informally assess the validity of data (Supplementary Table 2), CARIS compares data collected by midwives at booking with that collected by fetal cardiologists who also take a history; some discrepancies are noted e.g. maternal smoking and the more complete data are retained by CARIS. Severity of cardiovascular anomalies is based on the EUROCAT classification [9].

Statistical analysis

Adjusted odds ratios (aOR) were estimated for socio-demographic, maternal, infant, and intervention factors associated with infant death i.e. death which occurred in the first year after birth. The analysis was performed separately for: infants with any anomaly; those with isolated anomalies; those with multiple anomalies; and those with cardiovascular anomalies.

Infant mortality rate (IMR) was calculated as the number of infant deaths per 10,000 livebirths, where the baby dies before their first birthday. ONS denominator data for IMR was the total number of livebirths in Wales between 1998 and 2016. The modeling strategy aimed to identify the strongest risk factors for infant death. The risk factors explored were

Confidential: For Review Only

socio-demographic, maternal, infant, and intervention factors (Figure 2). *A priori* factors included maternal ethnicity, infant sex and gestational age at birth, which were selected based on evidence in the literature that they were important socio-demographic and clinical determinants of infant deaths [4-5, 11-12], and hence these variables were included in the final model regardless of their statistical significance in the univariable analysis. For the remaining variables, unadjusted odd ratios (uORs) were estimated in an univariable analysis to identify variables associated with death ($p<0.1$), which were then explored in a multivariable logistic regression model to generate adjusted odds ratios (aORs). Variables were dropped from the multivariable model using a backward stepwise approach if they did not significantly improve the fit of the data (i.e. $p<0.05$ in the likelihood ratio test) with examination of the results as each variable was removed. Interaction was tested between gestational age and other variables in the final model, $p<0.05$ was considered as statistically significant.

A sensitivity analysis was conducted for each subgroup by adding year of birth to the final model to investigate if there were any significant changes in the effect of key risk factors associated with infant death. In addition, for variables including maternal ethnicity, maternal smoking, history of anomalies in previous pregnancy, and infant surgery status, the amount of missing data was high (i.e. 15-41%) and associated with other variables (i.e. not missing completely at random). As it was not possible to exclude that the missingness may also have been at random, multiple imputation was justified and performed for these variables[36] using multiple imputation chained equations (MICE). Sensitivity analyses were conducted using complete case analysis and imputed data to check the robustness of results in the final models. All statistical analyses were performed using Stata 13 [13].

Confidential: For Review Only

Research ethics and statistical disclosure

The de-identified health data for this study was provided by the Secure Anonymised Information Linkage (SAIL) databank; research ethics committee approval was not required for this secondary data analysis study. This study was approved by the SAIL Information Governance Review Panel (IGRP). All statistical disclosure control and checks were strictly followed according to the SAIL Databank and ONS guidelines, to ensure that individual infants were not deductively identifiable.

Patient and public involvement

All proposals to use data within the SAIL Databank are subject to review by an independent Information Governance Review Panel (IGRP) for privacy risk, data governance and public benefit assessment. The IGRP is made up of a range of independent experts as well as members of the public.

Results

A total of 632,945 livebirths occurred to residents in Wales between 1998 and 2016. In this period, 30,424 infants affected by congenital anomalies were identified, of which 20,008 (65.8%) had isolated anomalies and 10,374 (34.1%) had multiple anomalies (Figure 1). There were 1,044 deaths of infants who were affected by either an isolated anomaly or multiple anomalies; this represented an IMR of 16.5 deaths per 10,000 livebirths and case fatality of 3.4% for infants with any anomaly. Infants with an anomaly who died represented almost one-third (30.3%) of all infant deaths (n = 3,443) in this period; about two-thirds (20.7%) of

Confidential: For Review Only

the anomaly related deaths were of infants with multiple anomalies and one-third (9.5%) were of infants with isolated anomalies (Table 1). Among all the isolated anomaly subgroups, cardiovascular anomalies were associated with the largest number of infant deaths (n = 84), representing an IMR of 1.33 deaths per 10,000 livebirths and case fatality of 2.7%.

Table 2 shows the characteristics of the infants with any anomalies who died compared with those who survived infancy. Compared with infants who survived, those who died were more likely to live in the most deprived areas (31% versus 28%), be girls (46% versus 39%), have a low birthweight (51% versus 15%), were born preterm (46% versus 15%), to have not received surgery (45% versus 40%), or had mothers who were of non-White ethnicity (9% versus 3%), multiparous (58% versus 50%), smokers (25% versus 17%), had a multiple pregnancy (11% versus 5%), and a maternal history of an anomaly in a previous pregnancy (11% versus 8%).

Table 3 shows the unadjusted odd ratios for variables associated with infant death ($p < 0.1$) by different anomaly subgroups. Birthweight and gestational age at birth were highly collinear (correlation coefficient $r = 0.75$). Only gestational age was therefore included in the final model, as it has greater clinical utility.

Table 4 shows that, for infants with any anomaly, significant increases in the adjusted odds of infant death were found among infants born to mothers who were of non-White ethnicity (aOR 2.25; 95% CI: 1.77-2.86); parous (aOR 1.24; 1.08-1.41); active smokers during pregnancy (aOR 1.20; 1.02-1.40); where the infant was born preterm (aOR 4.38; 3.86-4.98); and was a girl (aOR 1.28; 1.13-1.46). Infants who required surgery in the first year after birth had a lower odds of infant death than those who did not (aOR 0.80; 0.68-0.95). A similar

Confidential: For Review Only

pattern of aORs was seen for isolated anomalies (except that infant sex was not statistically significant) and multiple anomalies (except maternal smoking was not statistically significant). For cardiovascular anomalies, the main risk factors were preterm birth and severity (having a most severe CHD versus less CHD: aOR 229; 90.8-579). Having surgery had a protective effect (aOR 0.34; 0.15-0.75) for infants with a cardiovascular anomaly. The effects of these variables were not materially different in the sensitivity analyses when adjusted for year of birth (Supplementary Table 3) and using complete case analysis and imputation (data not shown). In addition, a more recent birth year was generally associated with a lower odds of death in infants with any congenital anomalies (aOR 0.96; 0.95-0.98 per yearly increase). There was no significant interaction between gestational age and other variables in the final model.

Discussion

This population-based cohort study used linked de-identified data from CARIS and ONS to investigate risk factors contributing to an excess risk of death of infants with congenital anomalies born to residents in Wales between 1998 and 2016. We found that infants with any congenital anomaly who died before their first birthday were more likely to be girls, be born preterm, in the earlier years of the cohort, not to have received surgery or have mothers who were of non-White ethnicity and smokers. Preterm birth was the strongest risk factor for excess infant deaths across all subgroups of congenital anomalies, but the effects of other factors on excess infant deaths varied according to the anomaly subgroup.

Previous studies have shown that significant inequalities in child health and IMR exist between ethnic groups in England and Wales, in which ethnic minority groups generally

Confidential: For Review Only

experience worse outcomes compared to White ethnic groups; however there is significant heterogeneity within most ethnic minority groups [14-15]. The excess deaths from congenital anomalies in minority ethnic groups is likely to be complex and may be due to an interplay of factors resulting in unequal access to and uptake of antenatal screening and medical and surgical interventions, different attitudes toward congenital anomalies and termination of pregnancy, consanguinity (e.g. a risk factor associated with more lethal anomalies) [16], as well as difference in genetics (e.g. incidence of genotype mutations), culture (e.g. attitude to healthcare and interventions), and behaviour (e.g. maternal smoking) between ethnic groups [4-5, 17-19].

The relationship between parity and infant mortality has been well documented although the exact mechanism is not clear; it is thought that biological including the impact of maternal age and sociological factors, as well as factors involving in accessing the health services may play a role [20-23]. Smoking during pregnancy is a well-known risk factor for intrauterine growth restriction, prematurity and fetal death. Previous studies have suggested that placental dysfunction and/or abruption lead to fetal hypoxia due to nicotine-induced vasoconstriction during the perinatal period [24-27]. In addition, smoking has been shown to be associated with an increased risk of sudden infant death syndrome [28]. It is possible that these effects are more serious for infants with an underlying anomaly. However, the literature on smoking in relation to congenital anomalies is not unequivocal. The study of Child Death Outcome Panel data in Bradford showed that smoking in pregnancy appeared protective against infant deaths from congenital anomalies, although the study population was small [29].

Confidential: For Review Only

Girls with a congenital anomaly had an increased risk of infant death compared to boys with anomalies overall, which is a finding not previous described. However, this is likely to be partially due a male excess of conditions such as pyloric stenosis and potentially hypospadias (not examined in this study), which are sex-specific and rarely lethal (Supplementary Table 4). Infants who are born preterm have an increased risk of co-morbidities and related complications such as intraventricular haemorrhage, chronic lung disease of prematurity and necrotising enterocolitis [30], and hence poorer prognostic outcomes in infancy in general compared with infants born at term. Having a major anomaly also increases the chance of an infant being born preterm [30-31]; potentially, both risk factors may contribute to the same chain of event leading to infant death.

The strong association found between infant mortality risk and severity of cardiovascular anomalies is to be expected. However, classification of disease severity among other anomaly subgroups are less well established, as the aetiology of many congenital anomalies is not known [32]. Advances in diagnostic, surgical and medical interventions have generally improved the survival of infants with congenital anomalies who require surgery; the majority of formally lethal anomalies can now be successfully treated [33]. Particularly, advances in cardiac surgery, imaging, prenatal screening and diagnosis have significantly reduced the risk of death for infants with a congenital heart defect over time [34]. However, death rates remain high for many severe anomalies in spite of medical and surgical interventions, for example, infants with the most severe cardiac conditions continue to experience a significant risk of post-operative cardiovascular sequelae and other complications [35]. We did not find an association between surgery and isolated anomalies, but it is possible that the prognosis of many less severe isolated anomalies is good regardless of surgery compared

Confidential: For Review Only

to multiple anomalies; however, further subgroup analysis of specific anomaly subtypes is needed to confirm this.

While our results were drawn from data from the period between 1998 and 2017, it is important to note that public health interventions such as the smoking cessation programme in Wales from 2007, and the Antenatal Screening Wales in 2003 may have had an impact on the mortality estimates, although assessing the impact of these programmes is outside the scope of this study. In our study, we found that the infant death rate tended to decrease over the study period for infants with any congenital anomalies.

The main strengths include the national study population and robust study design. The study cohort was identified from a high-quality, population-based congenital anomaly registry with an active surveillance system which covers all births in Wales. The multiple source reporting system maximises case finding and thus internal validity [36]. A further strength relates to the broad definition of congenital anomalies, which includes structural and chromosomal defects, as well as rare diseases of congenital origin. Consequently, we were able to generate robust epidemiological findings and assess the full impact of a wide group of congenital anomalies and rare diseases on infant death.

As the occurrence of anomaly-related infant deaths is rare, it was not possible to investigate risk factors for infant mortality for specific anomalies except for cardiovascular anomaly subgroup. Consequently, heterogeneity inevitably exists within anomaly groups in terms of infant mortality risk. In addition, the number of variables explored in this study was restricted by the data available, which is a common limitation of using routinely collected data. The extent of missing data in variables such as maternal ethnicity and smoking is

Confidential: For Review Only

significant, and self-reporting of smoking is not always reliable [37], therefore caution should be taken when interpreting these results. The odd ratios of anomaly severity associated with death of infants with a CHD have wide confidence intervals due to the small sample size; however, it was not appropriate to group the most severe and moderately severe CHD together, as this would lead to considerable heterogeneity. In addition, as the Welsh population is predominantly White (>95%), a more nuanced analysis by ethnic subgroups is not possible due to small numbers. Finally, some severe cardiac anomalies are not amenable to surgery, or parents do not wish to put their child through years of major surgical intervention and may instead opt for palliative care, thus potential selection bias cannot be excluded even though surgical intervention has improved over years.

Conclusions

Congenital anomalies are a leading cause of infant death. Socio-demographic, maternal, infant and interventional factors have a significant impact on death in infants with congenital anomalies by likely different potential mechanisms. Improving access to prenatal care, optimising care for preterm infants, smoking cessation advice and surgery may help lower the risk of infant death.

Confidential: For Review Only

What is known about the subject:

- Congenital anomalies are a leading cause of infant death.
- Evidence about the risk factors contributing to the death of infants with congenital anomalies is limited.

What this study adds:

- A third of infant deaths in Wales involved an infant with a congenital anomaly.
- Preterm birth was the strongest risk factor for excess infant deaths.
- Socioeconomic factors including maternal ethnicity and smoking are risk factors for excess infant deaths.

Confidential: For Review Only

Data sharing statement

The de-identified health data for this study was provided by the Secure Anonymised Information Linkage (SAIL) databank.

Acknowledgements

We thank the SAIL team for providing the datasets and their assistance with data linkage for this study, and the CARIS team for their support with the CARIS data.

Funding

This study is part-funded by the National Institute for Health Research (NIHR) Policy Research Programme, conducted through the NIHR Policy Research Unit in Maternal Health and Care, 108/0001. The views expressed are those of the author(s) and not necessarily those of the NIHR or the Department of Health and Social Care.

Peter Ho is funded by a Clarendon – Green Templeton College – Nuffield Department of Population Health Scholarship, University of Oxford.

Competing interests None declared.

Contributorship statement:

PH contributed to the design of the study, acquisition, analysis, interpretation of data, drafting and revising it critically, approved the final version to be published, and agrees to be accountable for all aspects of the work in ensuring that questions related to the accuracy or integrity of any part of the work are appropriately investigated and resolved.

MQ and JK contributed to the design of the study, acquisition, analysis, and interpretation of data for the work; supervised the project and revised it critically; approved the final version to be published; and agree to be accountable for all aspects of the work in ensuring that questions related to the accuracy or integrity of any part of the work are appropriately investigated and resolved.

DT contributed to the acquisition, interpretation of data for the study, revised it critically, approved the final version to be published, and agrees to be accountable for all aspects of the work in ensuring that questions related to the accuracy or integrity of any part of the work are appropriately investigated and resolved.

Confidential: For Review Only

Table 1: Infant mortality rate by congenital anomaly subgroup in Wales (1998-2016 birth cohort).

Anomaly subgroup[2]	Livebirths N = 632,945[1], (%)	Deaths (%) N = 3,443[2] (%)	Case fatality	IMR per 10,000 livebirths
All anomalies[3]	30,424 (4.8)	1,044 (30.3)	3.4%	16.5
All isolated anomalies	20,008 (3.2)	327 (9.5)	1.6%	5.17
All multiple anomalies	10,374 (1.6)	714 (20.7)	6.9%	11.3
Isolated cardiovascular anomalies	3,149 (0.5)	84 (2.4)	2.7%	1.33

[1]Total number of livebirths in Wales between 1998 and 2016 (ONS data) as the denominator. [2]Total number of deaths in Wales between 1998 and 2016 (ONS and CARIS data) as the denominator. [3]The number of isolated and multiple anomalies (reported in CARIS) does not sum to all anomalies due to coding issues of <0.1% of cases.

Confidential: For Review Only

Table 2: Descriptive characteristics of infants with any anomaly who died and those who survived in the first year after birth.

	Died in infancy, n (%)	Survival infancy, n (%)
Socio-demographic factor		
Townsend quintile		
1 (least deprived)	122 (12)	4,564 (16)
2	160 (15)	4,594 (16)
3	207 (20)	5,361 (19)
4	225 (22)	5,861 (21)
5 (most deprived)	327 (31)	7,886 (28)
Maternal ethnicity		
White	715 (69)	15,807 (56)
Other	89 (9)	902 (3)
Not known/ missing	240 (23)	11,646 (41)
Maternal age at birth, years *(n = 29,400)*		
Mean +/- SD (year)	*28.3 +/- 6.5*	*28.2 +/- 6.2*
≤ 24	318 (31)	8,514 (30)
25 – 29	288 (28)	7,835 (28)
30 – 34	239 (23)	7,129 (25)
≥ 35	199 (19)	4,849 (17)
Maternal factors		
Parity		
Nulliparous	385 (37)	11,115 (39)
≥ 1	606 (58)	14,116 (50)
Multiple pregnancy		
Yes	114 (11)	1,334 (5)
No	930 (89)	27,022 (95)
Maternal smoking		
Smoker	261 (25)	4,712 (17)
Non/ Ex smoker	541 (52)	12,110 (42)
Not known / missing	242 (23)	11,534 (41)
Anomalies in previous pregnancies		
Yes	117 (11)	2,257 (8)
No	724 (69)	16,153 (57)
Not known / missing	203 (19)	9,946 (35)
Infant factors		
Infant sex		
Male	566 (54)	17,810 (61)
Female	474 (46)	11,550 (39)
Birthweight, grams		
Median (IQR)	*2475 (1650-3150)*	*3250 (2780-3650)*
< 2500 (low birthweight)	529 (51)	4,505 (15)
≥ 2500	510 (49)	23,835 (81)
Gestational age, week		
Median (IQR)	*37 (32-39)*	*39 (38-40)*
< 37^{+0} (preterm)	480 (46)	4,510 (15)
≥ 37^{+0} (term)	562 (54)	23,935 (82)
Intervention factors		
Surgery		
Performed (or expected) in the first year after birth	216 (21)	6,405 (22)
Not performed or required in the first year after birth[2]	465 (45)	11,833 (40)
Not known/ missing	363 (35)	11,142 (38)

Category with small number is not shown due to risk of statistical disclosure. [2]Including a minority of conditions which were too severe for surgery. Note: Confirmed and probable cases were included in the analysis.

Confidential: For Review Only

Table 3: Unadjusted odds ratios for infant mortality by anomaly subgroup.

Factors	All anomalies	Isolated anomalies	Multiple anomalies	Cardiovascular anomalies
Townsend quintile				
1 (least deprived)	1 (reference)	1 (reference)	1 (reference)	1 (reference)
2	1.30 (1.03-1.65)	1.30 (0.86-1.95)	1.29 (0.96-1.73)	1.61 (0.76-3.41)
3	1.44 (1.15-1.81)	1.27 (0.85-1.89)	1.48 (1.12-1.96)	1.25 (0.59-2.66)
4	1.44 (1.15-1.80)	1.35 (0.92-2.00)	1.37 (1.04-1.80)	0.91 (0.41-2.01)
5 (most deprived)	1.55 (1.26-1.92)	1.46 (1.02-2.11)	1.56 (1.20-2.02)	1.33 (0.67-2.66)
Maternal ethnicity[1]				
White	1 (reference)	1 (reference)	1 (reference)	1 (reference)
Other	2.18 (1.73-2.75)	1.77 (1.11-2.82)	2.34 (1.78-3.08)	1.00 (0.31-3.26)
Not known/ missing	0.46 (0.39-0.53)	0.59 (0.47-0.75)	0.56 (0.46-0.68)	0.71 (0.44-1.13)
Maternal age at birth, years				
25-29	1 (reference)	1 (reference)	1 (reference)	1 (reference)
≤ 24	1.02 (0.86-1.20)	1.04 (0.79-1.37)	0.97 (0.79-1.19)	1.12 (0.64-1.96)
30-34	0.91 (0.77-1.09)	0.77 (0.56-1.05)	0.96 (0.78-1.19)	0.77 (0.41-1.45)
≥ 35	1.12 (0.93-1.34)	0.92 (0.65-1.28)	1.12 (0.90-1.41)	1.14 (0.60-2.18)
Parity				
Nulliparous	1 (reference)	1 (reference)	1 (reference)	1 (reference)
≥ 1	1.24 (1.09-1.41)	1.48 (1.16-1.89)	1.18 (1.01-1.39)	1.16 (0.72-1.85)
Not known/ missing	0.49 (0.37-0.65)	0.99 (0.67-1.46)	0.37 (0.23-0.61)	1.16 (0.52-2.56)
Multiple pregnancy				
No	1 (reference)	1 (reference)	1 (reference)	1 (reference)
Yes	2.48 (2.03-3.04)	2.57 (1.80-3.65)	2.35 (1.83-3.04)	1.40 (0.64-3.08)
Maternal smoking				
Non/ Ex smoker	1 (reference)	1 (reference)	1 (reference)	1 (reference)
Smoker	1.24 (1.07-1.44)	1.52 (1.15-2.01)	1.11 (0.92-1.33)	1.91 (1.12-3.25)
Not known / missing	0.47 (0.40-0.55)	0.66 (0.52-0.85)	0.57 (0.46-0.70)	0.85 (0.51-1.43)
Anomalies in previous pregnancies				
No	1 (reference)	1 (reference)	1 (reference)	1 (reference)
Yes	1.16 (0.95-1.41)	1.24 (0.86-1.79)	1.25 (0.98-1.59)	0.56 (0.20-1.56)
Not known / missing	0.46 (0.39-0.53)	0.70 (0.55-0.89)	0.53 (0.42-0.66)	0.96 (0.60-1.53)
Infant sex[1]				
Male	1 (reference)	1 (reference)	1 (reference)	1 (reference)
Female	1.29 (1.14-1.46)	1.08 (0.86-1.35)	1.33 (1.14-1.55)	0.79 (0.51-1.22)
Birthweight, grams[2]				
≥ 2500	1 (reference)	1 (reference)	1 (reference)	1 (reference)
< 2500 (low birthweight)	5.49 (4.84-6.22)	5.32 (4.26-6.64)	4.54 (3.88-5.30)	3.34 (2.13-5.23)
Gestational age at birth[1,2]				
$\geq 37^{+0}$ (term)	1 (reference)	1 (reference)	1 (reference)	1 (reference)
$< 37^{+0}$ (preterm)	4.53 (4.00-5.14)	4.72 (3.78-5.90)	3.84 (3.29-4.49)	2.76 (1.75-4.35)
CHD[3] anomaly severity				
Less severity	n/a	n/a	n/a	1 (reference)
Moderate severity				10.1 (4.9-21.1)
Most severity				90.7 (43.4-189)

Confidential: For Review Only

Table 3 (continue): Unadjusted odds ratios for infant mortality by anomaly subgroup.

Factors	All anomalies	Isolated anomalies	Multiple anomalies	Cardiovascular anomalies
Surgery				
Not performed or required in the first year after birth[5]	1 (reference)	1 (reference)	1 (reference)	1 (reference)
Performed (or expected) in the first year after birth	0.86 (0.73-1.01)	0.80 (0.58-1.10)	0.68 (0.56-0.82)	2.93 (1.66-5.17)
Not known/ missing	0.83 (0.72-0.95)	0.92 (0.73-1.17)	0.76 (0.64-0.91)	0.75 (0.44-1.26)

[1]a priori variable; [2]Gestational age at birth and birthweight are strongly correlated ($r \geq 0.7$). Note: Statistical significance at $p < 0.1$; [3]CHD = Congenital heart defects; NS = non-significant.

Confidential: For Review Only

Table 4: Adjusted odds ratios for factors associated with infant death by anomaly subgroup.

Factors	All anomalies	Isolated anomalies	Multiple anomalies	Cardiovascular anomalies
Ethnicity<br>Other vs. White	2.25 (1.77-2.86)	1.87 (1.16-3.02)	2.38 (1.79-3.16)	1.08 (0.29-4.04)
Parity<br>≥ 1 vs. Nulliparous	1.24 (1.08-1.41)	1.48 (1.16-1.89)	1.19 (1.02-1.40)	
Maternal smoking<br>Smoker vs. Non/Ex smoker	1.20 (1.02-1.40)	1.44 (1.07-1.92)		
Infant sex<br>Female vs. Male	1.28 (1.13-1.46)	1.06 (0.85-1.33)	1.37 (1.17-1.61)	1.26 (0.78-2.06)
Gestational age at birth<br>Preterm vs. Term	4.38 (3.86-4.98)	4.53 (3.62-5.67)	3.86 (3.30-4.53)	3.67 (2.19-6.16)
CHD severity<br>Moderate vs. Less<br>Most vs. Less	n/a	n/a	n/a	18.4 (8.10-41.7)<br>229 (90.8-579)
Surgery<br>Yes vs. No	0.80 (0.68-0.95)		0.71 (0.58-0.86)	0.34 (0.15-0.75)

Note: Statistical significance at $p < 0.05$; Adjusted odd ratios with 95% confidence intervals are shown. Empty cell represents variable that was not included in the multivariable analysis. CHD = Congenital heart defects. n/a = not applicable.

Confidential: For Review Only

References

1. European Surveillance of Congenital Anomalies (EUROCAT) 2019, *EUROCAT Network Overview: Congenital anomalies and public health,* EUROCAT, viewed 26 August 2019, <https://eu-rd-platform.jrc.ec.europa.eu/eurocat/eurocat-network/eurocat-network-overview_en#inline-nav-2>.

2. Public Health England (PHE) 2019, National Congenital Anomaly and Rare Disease Registration Service Congenital Anomaly Statistics 2017, PHE, viewed 8 September 2018, <https://assets.publishing.service.gov.uk/government/uploads/system/uploads/attachment_data/file/821449/NCARDRS_congenital_anomaly_stats_2017.pdf>.

3. Office for National Statistics (ONS) 2015, *Statistical bulletin: Birth Summary Tables, England and Wales: 2014,* ONS, viewed 8 September 2019, <https://www.ons.gov.uk/peoplepopulationandcommunity/birthsdeathsandmarriages/livebirths/bulletins/birthsummarytablesenglandandwales/2015-07-15>.

4. Bol KA, Collins JS, Kirby RS. Survival of infants with neural tube defects in the presence of folic acid fortification. *Pediatrics.* 2006Mar1;117(3):803-13.

5. Shin M, Kucik JE, Siffel et al., Improved survival among children with spina bifida in the United States. *The Journal of pediatrics.* 2012 Dec 1;161(6):1132-7.

6. Best KE, Rankin J. Long-term survival of individuals born with congenital heart disease: a systematic review and meta-analysis. *Journal of the American Heart Association*. 2016 Jun 16;5(6):e002846.

7. Clark RH, Walker MW, Gauderer MW. Factors associated with mortality in neonates with gastroschisis. *European Journal of Pediatric Surgery*. 2011 Jan;21(01):21-4.

8. Congenital Anomaly Register and Information Service (CARIS) 2018, *Introduction. Congenital Anomaly Register and information Service*, CARIS, viewed 8 March 2019, <http://www.caris.wales.nhs.uk/introduction>.

9. European Surveillance of Congenital Anomalies (EUROCAT) 2013, EUROCAT Guide 1.4: Instruction for the registration of congenital anomalies. EUROCAT Central Registry, University of Ulster. Last update version 28/12/2018, EUROCAT, viewed 25 August 2019, <https://eu-rd-platform.jrc.ec.europa.eu/sites/default/files/Full_Guide_1_4_version_28_DEC2018.pdf>.

Confidential: For Review Only

10. World Health Organization (WHO), 2004. ICD-10: international statistical classification of diseases and related health problems: tenth revision, WHO, viewed 25 August 2019, < https://www.who.int/standards/classifications/classification-of-diseases>.

11. Melamed N, Yogev Y, Glezerman M. Fetal gender and pregnancy outcome. *The Journal of Maternal-Fetal & Neonatal Medicine*. 2010 Apr 1;23(4):338-44.

12. Lisi A, Botto LD, Rittler M et al., Sex and congenital malformations: an international perspective. *American Journal of Medical Genetics Part A*. 2005 Apr 1;134(1):49-57.

13. StataCorp. Stata multiple-imputation reference manual. College Station, TX: Stata Press, 2013: 48.

14. Moser K, Stanfield KM, Leon DA. Birthweight and gestational age by ethnic group, England and Wales 2005: introducing new data on births. *Health Stat Q.* 2008 Jan 1;39(39):22-31.

15. Gray R, Headley J, Oakley L et al., Towards an understanding of variations in infant mortality rates between different ethnic groups in England and Wales. *National Primate Epidemiology Unit, University of Oxford, and Department of Public Health (November 2009).* 2009 Nov:1-9.

16. Shawky RM, Elsayed SM, Zaki ME et al., Consanguinity and its relevance to clinical genetics. *Egyptian Journal of Medical Human Genetics*. 2013 Apr 17;14(2).

17. Dominguez TP, Dunkel-Schetter C, Glynn LM et al., Racial differences in birth outcomes: the role of general, pregnancy, and racism stress. *Health psychology*. 2008 Mar;27(2):194.

18. Weightman AL, Morgan HE, Shepherd MA et al., Social inequality and infant health in the UK: systematic review and meta-analyses. *BMJ open.* 2012 Jan 1;2(3):e000964.

19. Botto LD, Yang Q. 5, 10-Methylenetetrahydrofolate reductase gene variants and congenital anomalies: a HuGE review. *American journal of epidemiology*. 2000 May 1;151(9):862-77.

20. Sonneveldt E, Plosky WD, Stover J. Linking high parity and maternal and child mortality: what is the impact of lower health services coverage among higher order births?. *BMC Public Health*. 2013 Sep;13(3):1-8.

21. Hook EB. The impact of aneuploidy upon public health: mortality and morbidity associated with human chromosome abnormalities. *InAneuploidy* 1985 (pp. 7-33).Springer,Boston,MA.

22. Cuckle HS. Primary prevention of Down's syndrome. *International journal of medical sciences.* 2005;2(3):93.

23. Hobcraft J, McDonald JW, Rutstein S. Child-spacing effects on infant and early child mortality. *Population Index.* 1983 Dec 1:585-618.

24. Rogers JM. Tobacco and pregnancy. *Reproductive Toxicology*. 2009 Sep 1;28(2):152-60.

25. Quinton AE, Cook CM, Peek MJ. The relationship between cigarette smoking, endothelial function and intrauterine growth restriction in human pregnancy. *BJOG: An International Journal of Obstetrics & Gynaecology*. 2008 May;115(6):780-4.

26. Tikkanen, M., 2011. Placental abruption: epidemiology, risk factors and consequences. *Acta obstetricia et gynecologica Scandinavica*, *90*(2), pp.140-149.

27. Andres RL, Day MC. Perinatal complications associated with maternal tobacco use. *InSeminars in Neonatology* 2000 Aug 1(Vol. 5, No. 3, pp. 231-241).WB Saunders.

28. Mitchell EA. Smoking: the next major and modifiable risk factor. Sudden infant death syndrome: New trends in the nineties. 1995:114-8.

29. Shipton D, Tappin DM, Vadiveloo T et al., Reliability of self reported smoking status by pregnant women for estimating smoking prevalence: a retrospective, cross sectional study. *Bmj.* 2009 Oct 30;339.

30. Butler AS, Behrman RE, editors. Preterm birth: causes, consequences, and prevention. *National academies press*; 2007 May 23.

31. Khoshnood B, Lelong N, Houyel L et al., Prevalence, timing of diagnosis and mortality of newborns with congenital heart defects: a population-based study. *Heart.* 2012 Nov 15;98(22):1667-73.

32. World Health Organization (WHO) 2020, *Congenital anomalies: causes,* WHO, viewed 29 April 2020, <https://www.who.int/health-topics/congenital-anomalies#tab=tab_2>.

Confidential: For Review Only

33. Farmer D, Sitkin N, Lofberg K et al., Surgical interventions for congenital anomalies. Essential Surgery: Disease Control Priorities. *The International Bank for Reconstruction and Development. Washington, DC: The World Bank.* 2015 Mar 23:129-49.

34. Holst KA, Said SM, Nelson TJ et al., Current interventional and surgical management of congenital heart disease: specific focus on valvular disease and cardiac arrhythmias. *Circulation research.* 2017 Mar 17;120(6):1027-44.

35. McCracken C, Spector LG, Menk JS et al., Mortality following pediatric congenital heart surgery: an analysis of the causes of death derived from the national death index. *Journal of the American Heart Association.* 2018 Nov 20;7(22):e010624.

36. Congenital Anomaly Register and Information Service (CARIS) 2020a, *Introduction: Reporting to CARIS*, CARIS, viewed 12 March 2020, <http://www.caris.wales.nhs.uk/reporting-to-caris>.

37. Hughes RA, Heron J, Sterne JA et al., Accounting for missing data in statistical analyses: multiple imputation is not always the answer. *International journal of epidemiology.* 2019 Aug 1;48(4):1294-304.

Confidential: For Review Only

Figure 1: Anomaly status and categories included in this study; births in Wales 1998 to 2016.

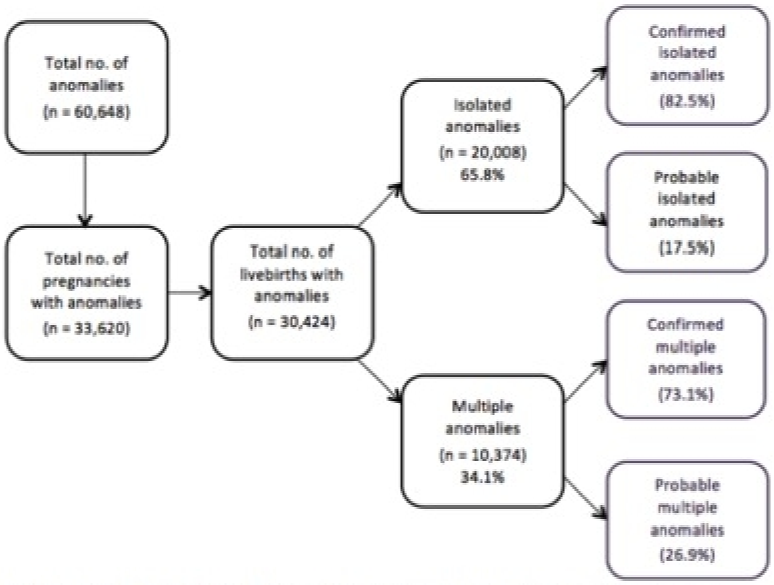

Note: The number of isolated and multiple anomalies does not sum to all anomalies due to coding issues of <0.1% of cases.

146x136mm (72 x 72 DPI)

Figure 2: Choices of the included confounding factors associated with infant death using a directed acyclic diagram (D.A.G.)

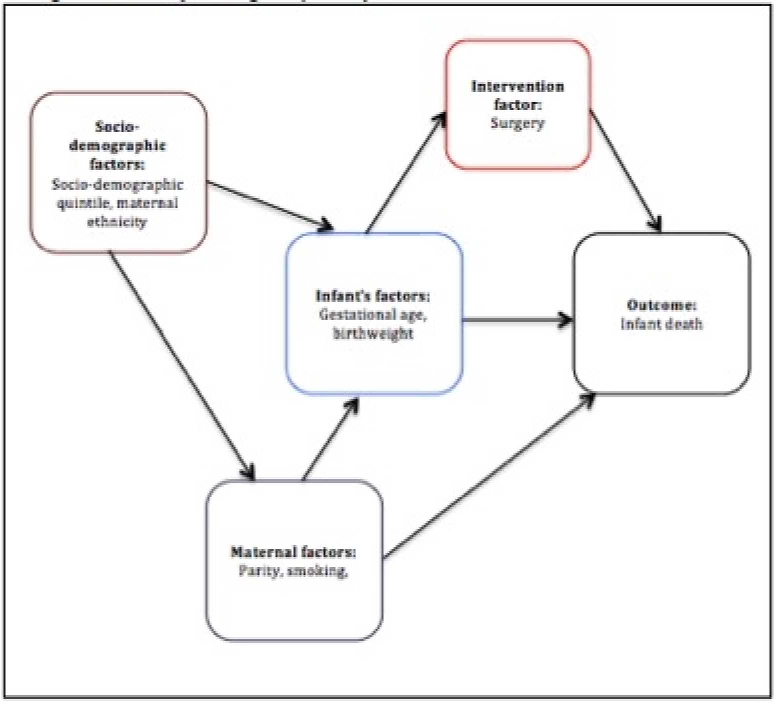

146x144mm (72 x 72 DPI)

Supplementary Table 1: Inclusion of anomaly subgroups.

Subgroup	Classification of anomaly included
All anomalies; all isolated anomalies; and all multiple anomalies	• Nervous system;<br>• Eye, ear, face and neck;<br>• Cardiovascular system;<br>• Respiratory and clefts;<br>• Abdominal wall defects and diaphragmatic hernia;<br>• Upper and lower gastrointestinal;<br>• Genitourinary;<br>• Limb and skeletal;<br>• Blood disorders;<br>• Endocrine and Metabolic;<br>• Neoplasm;<br>• Syndromes and congenital malformation syndromes;<br>• Maternal Infection leading to malformations.
Cardiovascular anomalies	• Congenital heart defects and diseases of the circulatory system.

Confidential: For Review Only

Supplementary Table 2: Description of included variables in the analyses.

Variable	Categorisation
Townsend (as area-based deprivation)	1 (least deprived)[1], 2, 3, 4, 5 (most deprived), and not known/missing.
Maternal ethnicity	White[2], Other (Chinese, Indian, Bangladeshi, Pakistani, Other Asian, Black Caribbean, Black African, Other Black, Mixed, Other), and not known/missing.
Maternal age at birth	≤24, 25-29[2], 30-34, ≥35 years, and not known/missing.
Parity	Nulliparous[2], ≥1, and not known/missing.
Multiple pregnancies	Yes, No[2], and not known/missing.
Maternal smoking	Smoker, Non/ Ex-smoker[2], and not known/missing.
Anomaly in previous pregnancies	Yes, No[2], and not known/missing.
Infant sex	Male, Female[2] and not known/missing.
Infant's birthweight	<2500 (low birthweight), ≥2500[2] g and not known/missing.
Infant's gestational age at birth[2]	$<37^{+0}$ (preterm), $\geq 37^{+0\,(1)}$, and not known/missing.
Disease severity (for CHD only)[3]	Less severity[1], moderate severity, most severity, and not known/missing.
Surgery	Performed (or expected) in the first year after birth, not performed or required in the first year after birth[1], and not known/missing.

[1]Reference group. [2]Significant departure of linearity was shown in the effect of gestational age in weeks on infant mortality.[3]Congenital heart defects (CHD) severity category was based on criteria used by Khoshnood et al. (2012): CHD less severe = Ventricular septal defect (VSD), Atrial septal defect (ASD), Pulmonary valve stenosis. CHD moderate severe = Common arterial truncus, Transposition of great vessels, Atrio-ventricular septal defect (AVSD), Tetralogy of Fallot, Pulmonary valve atresia, Aortic valve atresia/ stenosis, Coarctation of aorta, total anomalous pulmonary venous return. CHD most severe = Single ventricle, Tricuspid atresia and stenosis, Ebstein's anomaly, Hypoplastic left heart and Hypoplastic right heart.

Confidential: For Review Only

Supplementary Table 3: Year of birth added as a covariate in the final model as a sensitivity analysis.

Factors	All anomalies	Isolated anomalies	Multiple anomalies	Cardiovascular anomalies
Ethnicity<br>Other vs. White	2.31 (1.82-2.94)	1.90 (1.18-3.07)	2.24 (1.76-2.84)	1.02 (0.27-3.89)
Parity<br>≥ 1 vs. Nulliparous	1.22 (1.07-1.40)	1.47 (1.15-1.88)	1.22 (1.08-1.40)	
Maternal smoking<br>Smoker vs. Non/Ex smoker	1.19 (1.02-1.39)	1.43 (1.07-1.91)		
Infant sex<br>Female vs. Male	1.28 (1.13-1.45)	1.05 (0.84-1.32)	1.28 (1.12-1.45)	1.23 (0.76-2.02)
Gestational age at birth<br>Preterm vs. Term	4.35 (3.83-4.94)	4.51 (3.59-5.64)	4.39 (3.87-4.99)	3.67 (2.18-6.17)
CHD severity<br>Moderate vs. Less<br>Most vs. Less	n/a	n/a	n/a	18.4 (8.10-41.7)<br>242 (94-620)
Surgery<br>Yes vs. No	0.78 (0.66-0.93)		0.68 (0.58-0.79)	0.31 (0.14-0.70)
Year<br>Per yearly increase (1998-2016)	0.96 (0.95-0.98)	0.98 (0.95-1.00)	0.96 (0.95-0.97)	0.93 (0.88-0.99)

Note: Statistical significance at p < 0.05; Adjusted odd ratios with 95% confidence intervals are shown. Empty cell represents variable that was not included in the multivariable analysis. CHD = Congenital heart defects. n/a = not applicable.

Confidential: For Review Only
